# Endothelial cell-specific expression of serine/threonine kinase 11 modulates dendritic cell differentiation

**DOI:** 10.1038/s41467-022-28316-6

**Published:** 2022-02-03

**Authors:** Qiang Zhao, Young-Min Han, Ping Song, Zhixue Liu, Zuyi Yuan, Ming-Hui Zou

**Affiliations:** 1 Center for Molecular and Translational Medicine, Georgia State University, Atlanta, GA 30303 USA; 2 Department of Cardiology, First Affiliated Hospital of Xi’an Jiaotong University, Xi’an, China

**Keywords:** Haematopoietic cell growth factors, Innate immunity, Growth factor signalling, Myelopoiesis

## Abstract

In the bone marrow, classical and plasmacytoid dendritic cells (DC) develop from the macrophage-DC precursor (MDP) through a common DC precursor (CDP) step. This developmental process receives essential input from the niche in which it takes place, containing endothelial cells (EC) among other cell types. Here we show that targeted deletion of serine/threonine kinase 11 (*Stk11*) encoding tumor suppressor liver kinase b1 (Lkb1) in mouse ECs but not DCs, results in disrupted differentiation of MDPs to CDPs, severe reduction in mature DC numbers and spontaneous tumorigenesis. In wild type ECs, Lkb1 phosphorylates polypyrimidine tract binding protein 1 (Ptbp1) at threonine 138, which regulates stem cell factor (*Scf*) pre-mRNA splicing. In the absence of Lkb1, exon 6 of *Scf* is spliced out, leading to the loss of Scf secretion. Adeno-associated-virus-mediated delivery of genes encoding either soluble Scf or the phosphomimetic mutant Ptbp1^T138E^ proteins rescued the defects of MDP to CDP differentiation and DC shortage in the endothelium specific *Stk11* knockout mice. In summary, endothelial *Stk11* expression regulates DC differentiation via modulation of *Scf* splicing, marking the *Stk11*-soluble-Scf axis as a potential cause of DC deficiency syndromes.

## Abstract (web summary)

In the bone marrow, dendritic cell development is governed by supporting cells, such as endothelial cells. Here authors show that expression of serine/threonine kinase 11 in endothelial cells regulates differentiation of dendritic cell precursors via modulating secretion of stem cell factor.

## Introduction

Dendritic cells (DC) are a major type of antigen-presenting cell. DCs effectively link the innate with the adaptive immune systems by inducing the activation and differentiation of various immune effector cells, such as naïve B and T lymphocytes^1,2^. These unique properties place DCs at the center of immune homeostasis^3,4^. Insufficient numbers of DCs lead to defective or dysregulated tumor immunosurveillance, or conversely, attack self-antigens to induce tissue inflammation and autoimmune disease^5,6^. A better understanding of the intrinsic and extrinsic cellular mechanisms regulating DC differentiation will identify new therapeutic targets for both cancer and autoimmune diseases.

DC developed from macrophage-DC precursor (MDP) into common DC precursor (CDP), then to pre-classical DCs that finally give rise to classical DCs and plasmacytoid DCs^7–9^. In mouse bone marrow, DCs and monocytes share a common progenitor, the MDP, whereas a distinct progenitor, the CDP, is dedicated to DC production^7^. Although this cascade of DC progenitors has been identified, it remains unknown if the bone marrow niche regulates DC differentiation. Bone marrow niche is a local perivascular microenvironment, created mainly by endothelial cells (ECs) and mesenchymal stromal cells, maintaining or regulating hematopoietic stem cell (HSC) and hematopoietic progenitor cell (HPC) development and function^10^. Particular HSCs or HPCs may occupy distinct bone marrow niches in vivo^11^, as most HSCs lie in direct contact with bone marrow microvessels, whereas the HPCs are less likely than HSCs to be immediately adjacent to sinusoidal endothelium^12–15^. Although extensive work has been carried out to identify and characterize the perivascular niche of HSC quiescence and self-renewal^10,16,17^, it remains unknown if the bone marrow niche regulates the differentiation of hematopoietic DC precursors, such as of the MDP-to-CDP, and how disorders of the bone marrow niche function trigger DC dysfunction-related diseases.

Stem cell factor (Scf; also known as KitL) is a key cytokine in hematopoiesis and binds to the c-Kit tyrosine receptor kinase^18–20^. ECs and leptin receptor-expressing perivascular stromal cells are the main sources of Scf required for hematopoiesis in normal mouse bone marrow^17^. There are two isoforms of Scf, membrane-bound and soluble, generated by alternative mRNA splicing^21^. Membrane-bound Scf seems to be much more important for HSC maintenance and self-renewal, as HSCs are depleted in compounds of steel and steel Dickie mutant mice (*Kitl*^Sl^/*Kitl*^Sl-d^; also called *Sl/Sl*^*d*^)^22,23^, which express normal levels of soluble Scf but lack the membrane-bound form^24^. In contrast, soluble Scf is more important for the normal development of restricted myeloid lineage^25^. Although recent data demonstrate that Scf is strictly required for the long-term expansion of DC precursors^26^, and c-Kit inhibitor fully blocks maturation of DCs from progenitors^27,28^, how Scf regulates the proliferation and retention of the restricted DC progenitor population in the bone marrow is unknown.

Serine/threonine kinase 11 (*Stk11*), which encodes liver kinase b1 (Lkb1), is a tumor suppressor that links energy metabolism with cell growth and proliferation^29,30^. *Stk11* is required for HSC maintenance^31–33^. Previous studies showed that constitutive deletion of *Stk11* in mice is embryonic lethal^34^. Loss of *Stk11* in DCs leads to aberrant DC antigen-presenting and T-cell homeostasis^35–37^. Our recent work indicated that endothelial *Stk11* maintains EC function via modulation of endothelial nitric oxide synthase (eNOS) expression^38,39^. In addition, *Stk11* deletion promotes angiogenesis through inhibited Rab7-dependent neuropilin-1 degradation and Sp1-mediated vascular endothelial growth factor (VEGF) expression^39,40^. However, whether *Stk11* in bone marrow ECs regulates *Scf* alternative splicing and DC development remains unclear.

Here, we report that *Stk11*^ec−/−^ mice spontaneously develop tumors with splenomegaly and disorganized immune activation characterized by decreased number of DCs and myeloid proliferation. Mechanistically, Lkb1 phosphorylates Ptbp1 to promote *Scf* exon 6 retention; thus, switches alternative splicing of *Scf* mRNA to favor soluble Scf, which regulates DC differentiation through cell-detached means of communication between bone marrow ECs and DC precursors in vivo.

## Results

### Endothelial cell-specific *Stk11-*deleted mice **(***Stk11*^ec−/−^**)** spontaneously develop tumors

**Fig. 1:**
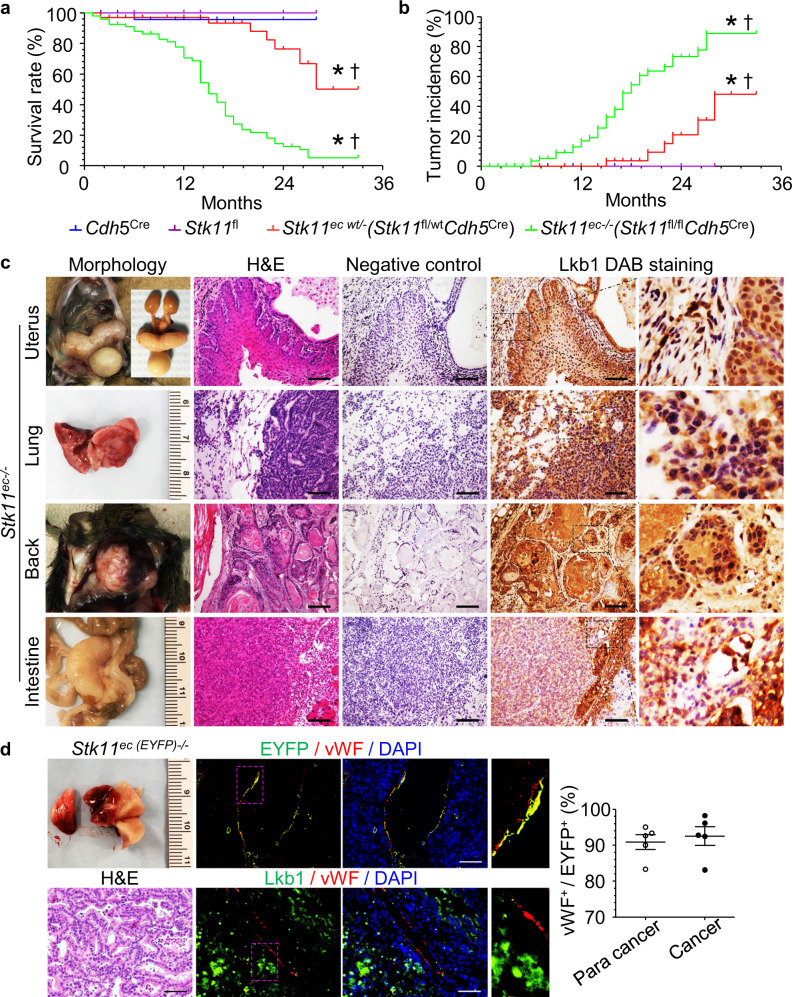
Endothelial cell (EC)-specific *Stk11* deleted (*Stk11*^ec−/−^) mice show spontaneous tumorigenesis in vivo. Survival rate (**a**) and tumor incidence (**b**) of *Cdh5*^Cre^ (*n* = 34), *Stk11*^fl^ (*n* = 17), *Stk11*^fl/wt^*Cdh5*^Cre^ (*n* = 65), and *Stk11*^fl/fl^*Cdh5*^Cre^ (*n* = 98) mice. ^*^P < 0.001 versus *Cdh5*^Cre^ and ^†^*P* < 0.001 versus *Stk11*^fl^ by log rank test (two-sided). **c** Representative images of gross anatomy, hematoxylin and eosin (H&E)-stained and Lkb1 DAB-stained tumor sections in *Stk11*^ec−/−^ mice. Scale bar: 50 μm. Images are representative of three independent experiments. **d** Representative images of lung tumor section from *Stk11*^ec(EYFP)−/−^ mice with EYFP/Lkb1(green), von Willebrand factor (vWF, red) and DAPI (blue)-staining. Scale bar: 50 μm. Bar graph summarizes the percentage of vWF^+^ (used as an EC marker) cells in EYFP^+^ cells.

Endothelial cell (EC)-specific *Stk11-*deleted (*Stk11*^ec−/−^*, Stk11*^fl/fl^*Cdh5*^Cre^) mice were generated as described previously^38^. Over time, natural death (found dead or moribund and euthanized) in *Stk11*^ec−/−^ mice occurred and was frequently accompanied by clinical signs attributable to tumor burden, including weight loss, dyspnea, or observable masses (Fig. 1a and Supplementary Table 1). The homozygous *Stk11*^ec−/−^ mice (*Stk11*^fl/fl^*Cdh5*^Cre^) developed tumors as early as 6 months of age, and heterozygous mice (*Stk11*^fl/wt^*Cdh5*^Cre^) developed tumors beginning at 15 months **(**Fig. 1b, Supplementary Tables 1 and 2). Hematoxylin-and-eosin (H&E) staining and histopathological examination further confirmed tumor development with different pathological diagnoses, such as carcinoma, adenocarcinoma, lymphoma, leukemia, and endometrial carcinoma (Fig. 1c). Tumor lesions were observed in multiple tissues of *Stk11*^ec−/−^ mice, including lung, uterus, kidney, ureter, abdominal cavity, bone, and lymph node (Supplementary Table 3). The overall incidence of tumor-bearing mice increased from 19.12% at 14 months of age, to 73.91% at 24 months of age in *Stk11*^ec−/−^ mice (Fig. 1b and Supplementary Table 3). The most common neoplasm at 14 months of age found in *Stk11*^ec−/−^ mice was an abdominal tumor (7.35%), and the most common neoplasm at 24 months and 30 months of age was lung tumor (21.74% and 14.29%, respectively) (Supplementary Table 3).

To explore whether tumors developed in *Stk11*^ec−/−^ mice originated from *Stk11* deleted ECs, we performed immunochemical staining of Lkb1 (encoded by *Stk11*) in tumor sections from *Stk11*^ec−/−^ mice. As shown in Fig. 1c, Lkb1 expression in cancer cells and cells in para-cancer areas were comparable (both Lkb1 positive). To validate this observation, we further generated *Stk11*^ec (EYFP) −/−^ mice (*Stk11*^fl/fl^*ROSA*^EYFP^*Cdh5*^cre^) to explore tumor origin and biology in vivo. In these mice, *Stk11*-depleted *Cdh5*^+^ cells and their progeny were irreversibly labeled with an enhanced yellow fluorescent protein (EYFP). In-depth microscopic analysis revealed that ECs were abundantly labeled with EYFP (>90%), whereas EYFP staining in non-ECs in the spontaneously developed tumor from *Stk11*^ec (EYFP)−/−^ mice was negligible (Fig. 1d). Given the evidence that tumor cells are Lkb1-positive and strict EYFP expression in tumor ECs (identified by positive staining for vWF [vWF^+^]) in the lineage-tracking mice (*Stk11*^ec (EYFP)−/−^), we concluded a non-cell-autonomous effect of EC *Stk11* deficiency on tumorigenesis or tumor suppression.

### *Stk11*^ec−/−^ mice exhibit disorganized immune activation

**Fig. 2:**
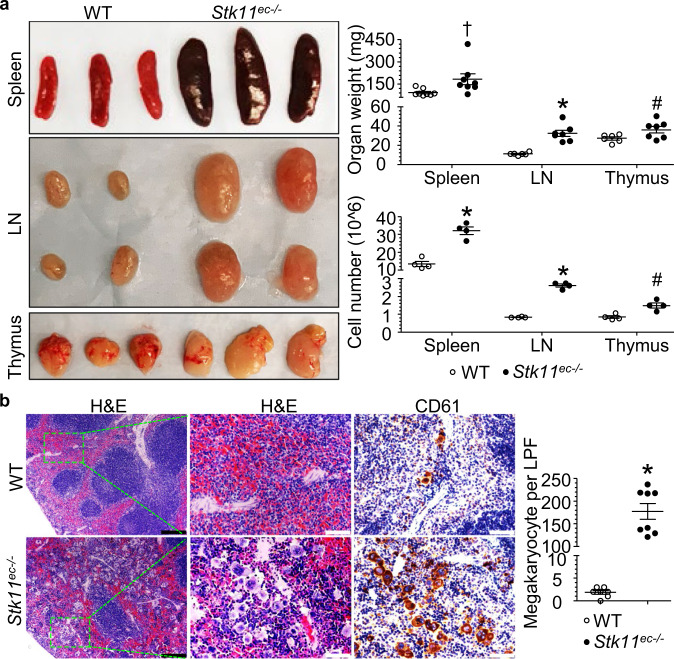
*Stk11*^ec−/−^ mice show disorganized immune activation in vivo. **a** Representative spleen, lymph node (LN) and thymus images and quantification of spleen, LN and thymus weight and cell number in WT and *Stk11*^ec−/−^ mice (12-weeks-old, mixed-gender, *n* = 6–8 in organ weight; *n* = 4 in cell number count). Organ weight: ^†^*P* = 0.03; ^*^P < 0.001; ^#^*P* = 0.04 versus WT; Cell number: ^*^P < 0.001; ^#^*P* = 0.008 versus WT analyzed by Student’s *t*-test (two-sided). **b** Representative images of H&E-stained (left) and CD61 (megakaryocyte marker) DAB-stained (right) spleen of WT and *Stk11*^ec−/−^ mice. Quantification of spleen megakaryocytes number per low power field (per LPF, 10× field) of WT and *Stk11*^ec−/−^ mice (12-weeks-old, mixed-gender, *n* = 7–8 each group). Scale bar: (Black) 200 μm, (White) 50 μm. *P < 0.001 versus WT analyzed by Student’s *t*-test (two-sided).

In addition to spontaneous tumor development in *Stk11*^ec−/−^ mice, spleens of 12-week-old *Stk11*^ec−/−^ mice weighed approximately three times more than those of littermate controls (Fig. 2a). Correlated with increased organ size and weight, the number of whole spleen cells in *Stk11*^ec−/−^ mice was significantly higher than that in littermate controls (Fig. 2a). Increased number of spleen cells was associated with extensive megakaryocyte infiltration (identified by positive staining for CD61 [CD61^+^]) in spleen red pulp (Fig. 2b). The *Stk11*^ec−/−^ mice also exhibited lymphadenopathy and enlarged thymus (Fig. 2a). Histopathological analysis revealed massive lymphocyte infiltration into multiple organs, including intestine, liver, pancreas, and lung in *Stk11*^ec−/−^ mice (Supplementary Fig. 1). Overall, these results indicate disorganized immune activation in *Stk11*^ec−/−^ mice.

### *Stk11*^ec−/−^ mice develop myeloid proliferative disorder with depressed DC differentiation

**Fig. 3:**
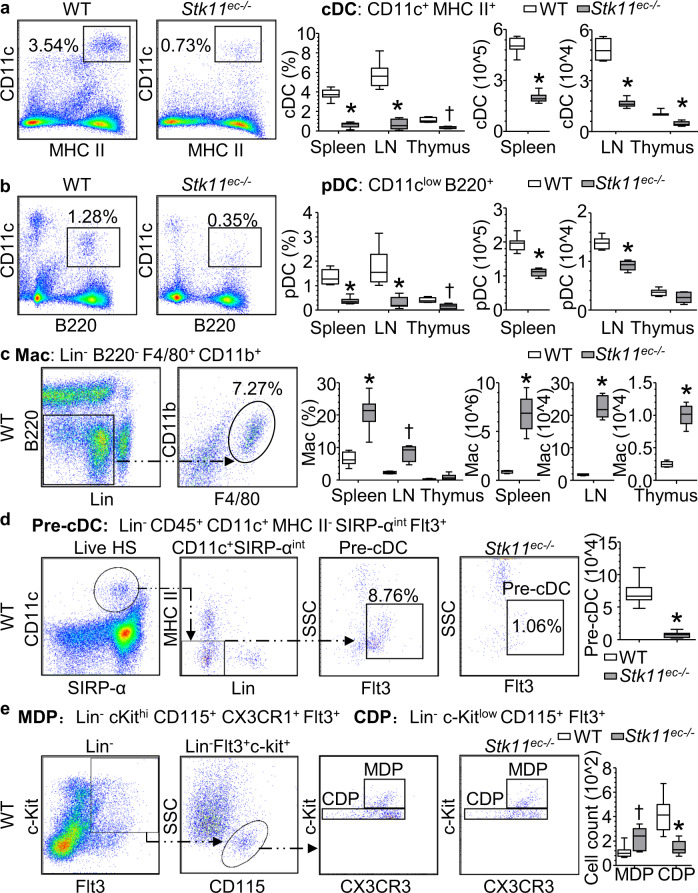
*Stk11*^ec−/−^ mice develop a myeloid proliferative disorder with depressed DC differentiation. **a**, **b** Flow cytometry analysis of spleen, lymph node (LN), and thymus cells isolated from WT or *Stk11*^ec−/−^ mice and stained for CD11c, MHC II, and B220. Classical DCs (cDCs) were defined as CD11c^+^ MHC II^+^, plasmacytoid DCs (pDCs) were defined as CD11c^low^ B220^+^. Bar graph summarizes cell number of cDC and pDC in spleen (*n* = 11–13 each group), LN (*n* = 11 each group), and thymus (*n* = 4–5 each group) (12-weeks-old, mixed-gender). ^†^*P* = 0.006; ^*^P < 0.001 versus WT by nonparametric Mann–Whitney *U* test (two-sided). **c** Flow cytometry analysis of spleen cells isolated from WT or *Stk11*^ec−/−^ mice and stained for Lin (CD3, CD19, CD49b, Ly6G), B220, F4/80, and CD11b. Macrophages were defined as Lin^-^ B220^−^ F4/80^+^ CD11b^+^. Bar graph summarizes cell number of macrophage in spleen (*n* = 11–13 each group), LN (*n* = 4–5 each group), and thymus (*n* = 4–6 each group) (12-weeks-old, mixed-gender). ^†^*P* = 0.003; ^*^P < 0.001 versus WT by nonparametric Mann–Whitney *U* test (two-sided). **d** Flow cytometry analysis of bone marrow cells isolated from WT or *Stk11*^ec−/−^ mice and stained for Lin (CD3, CD19, Ter119, NK1.1, B220), CD45, CD11c, MHC II, SIRP-α, and Flt3. Committed precursors of cDC (pre-cDC) were defined as Lin^-^ CD45^+^ CD11c^+^ MHC II^−^ SIRP-^αint^ Flt3^+^. Bar graph summarizes cell number of pre-cDC in bone marrow (12-weeks-old, mixed-gender, *n* = 12–13 each group). *P < 0.001 versus WT by nonparametric Mann–Whitney *U* test (two-sided). **e** Flow cytometry analysis of bone marrow cells isolated from WT or *Stk11*^ec−/−^ mice and stained for Lin, c-Kit, CD115, CX3CR1, and Flt3. Macrophages and DC precursors (MDPs) were defined as Lin^−^ c-Kit^high^ CD115^+^ CX3CR1^+^ Flt3^+^. Common DC precursors (CDPs) were defined as Lin^−^ c-Kit^low^ CD115^+^ Flt3^+^. Bar graph summarizes cell number of MDP/CDP in bone marrow (12-weeks-old, mixed-gender, *n* = 9–13 each group). ^†^*P* = 0.002; ^*^P < 0.001 versus WT by nonparametric Mann–Whitney *U* test (two-sided).

Regarding our evidence of disorganized immune equilibrium in *Stk11*^ec−/−^ mice, we next examined the fate and distribution of immune cells in *Stk11*^ec−/−^ and littermate control mice. Flow cytometry analysis showed the numbers of both classical DCs (CD11c^+^ MHC II^+^), plasmacytoid DCs (CD11c^low^ B220^+^), DC1 (CD103^+^ CD11b^−^) and DC2 (CD103^−^ CD11b^+^ CD64^−^) in the spleen, lymph node, and thymus were significantly lower in *Stk11*^ec−/−^ mice than their littermate controls (Fig. 3a, b, Supplementary Fig. 2a). Conversely, the numbers of granulocytes (Lin^−^ CD45^+^ FSC^high^ SSC^high^) and macrophages (Lin^−^ B220^−^ F4/80^+^ CD11b^+^) were higher in *Stk11*^ec−/−^ mice (Supplementary Fig. 2b, Fig. 3c) than their littermate controls.

### Differentiation of MDP into CDP is suppressed in *Stk11*^ec−/−^ mice

Next, we sought to identify the key reason for DC depression and myeloid proliferation in *Stk11*^ec−/−^ mice. Firstly, we checked the number of DC progenitors in bone marrow (pre-classical DCs: Lin^−^ CD45^+^ CD11c^+^ MHC II^-^ SIRP-α^int^ Flt3^+^; MDP: Lin^−^ c-Kit^high^ CD115^+^ CX3CR1^+^ Flt3^+^; CDP: Lin^−^ c-Kit^low^ CD115^+^ Flt3^+^). The number of pre-classical DC (Pre-cDC) and CDP showed an 80% reduction (Fig. 3d, e), whereas the number of MDP increased (Fig. 3e). We also noticed that there was no significant difference in the number of HSC, multipotent progenitor (MPP), lymphoid-primed multipotent progenitor (LMPP), common lymphoid progenitor (CLP), common myeloid progenitor (CMP), and granulocyte-monocyte progenitor (GMP) between *Stk11*^ec−/−^ mice and littermate control mice (Supplementary Fig. 3). Taken together, these data suggest that *Stk11*^ec−/−^ mice underwent a myeloid shift toward macrophages, monocytes, and granulocytes, associated with depressed DC lineage and MDP-to-CDP differentiation, independent of HSC or other hematopoietic progenitor cell (HPC) maintenance and differentiation (Supplementary Fig. 4).

Next, we sought to determine whether *Stk11* deletion in ECs regulates terminal differentiated DC function. To this end, we challenged *Stk11*^ec−/−^ mice with B16 tumors expressing the model antigen ovalbumin (B16-OVA). Compared with WT mice, tumor growth was significantly increased in *Stk11*^ec−/−^ (Supplementary Fig. 5a). Immunization of DCs from either WT or *Stk11*^ec−/−^ mice at same dose delayed B16 tumor growth in *Stk11*^ec−/−^ mice (Supplementary Fig. 5a). Additionally, B16-OVA bearing *Stk11*^ec−/−^ mice immunized with either WT or *Stk11*^ec−/−^ derived DCs showed similar OVA epitope (SIINFEKL) complex expression (Supplementary Fig. 5b). Altogether, these results demonstrated that *Stk11* deletion in ECs does not affect the function of terminal differentiated DCs.

### Selective EC-deletion of *Stk11* in adult mice also show obstructed MDP-to-CDP differentiation

Recent studies proposed that HSC and EC progenitors originate from a common mesodermal precursor^41,42^. These populations diverge before the onset of definitive hematopoiesis. After the onset of definitive hematopoiesis, Cdh5 (VE-cadherin) is considered a specific marker of ECs^43^. In this regard, we checked whether *Cdh5*-Cre recombinase leaked to DCs in vivo. As showed in Supplementary Fig. 6, *Cdh5*-Cre driven *Stk11* exon 3 to exon 6 excision mainly happens in ECs, not DCs.

Additionally, we generated tamoxifen-inducible *Stk11*^ec−/−^ mice by cross-breeding *Stk11*^fl/fl^ mice with VECad-Cre^ERT2^ mice^44^ and deleted *Stk11* in ECs at the age of 12 weeks. Similar to the constitutive *Stk11*^ec−/−^ mice, inducible EC-*Stk11* deletion in adult mice also showed enlarged spleen, lymph node, and thymus (Supplementary Fig. 7a), associated with extensive megakaryocyte infiltration in the spleen red pulp compared with littermate control mice (Supplementary Fig. 7b). Consistent with this observation, inducible EC-*Stk11* deletion in adult mice also showed depressed DC lineage development, myeloid shift towards macrophages, and significant higher MDP/CDP ratio (Supplementary Fig. 7c–e).

### Selective DC-deletion of *Stk11* in mice does not affect MDP-to-CDP differentiation

To further determine the relationship between *Stk11* and DC differentiation, we generated *Stk11*^fl/fl^*Cx3cr1*^cre^ mice (*Stk11*^dc−/−^) to delete *Stk11* in DC lineage^45^. Interestingly, the size of spleen, lymph node, and thymus showed no significant difference between *Stk11*^dc−/−^ mice and littermate control mice (Supplementary Fig. 8a). Moreover, the spleen structure of *Stk11*^dc−/−^ mice is comparable to littermate control mice, without accumulated megakaryocyte infiltration in spleen red pulp (Supplementary Fig. 8b). Importantly, depletion of *Stk11* in DC lineage does not affect numbers of classical DCs, plasmacytoid DCs, macrophages, or the MDP/CDP ratio in vivo (Supplementary Fig. 8c–e). Taken together, we conclude that selective deletion of *Stk11* in DC does not affect MDP-to-CDP differentiation.

### Cell-detached means of communication between bone marrow ECs and DC precursors in vivo

**Fig. 4:**
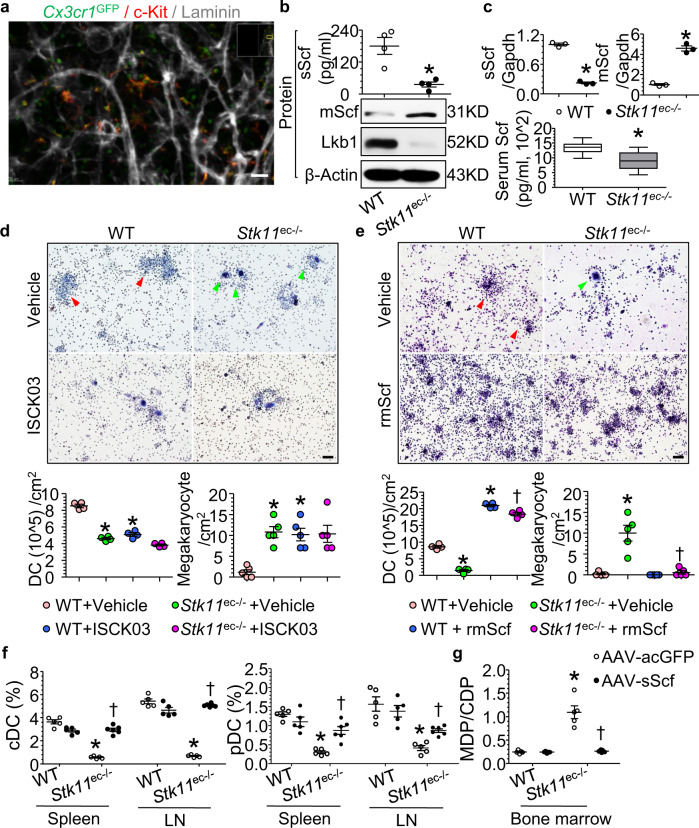
*Stk11* in ECs regulates soluble stem cell factor (sScf) to drive DC differentiation. **a** Deep imaging of *Cx3cr1*-GFP^+^ cells (green), hematopoietic progenitors (c-Kit^+^, red), and blood vessels (laminin, gray) in digitally reconstructed bone marrow (300 μm thick). Scale bar: 20 μm. Images are representative of three independent experiments. **b**, **c** Supernatant of cultured primary bone marrow ECs from WT or *Stk11*^ec−/−^ mice was subjected to soluble Scf (sScf) ELISA (b, upper) (*n* = 4 each group). *P = 0.005 versus WT by Student’s *t*-test (two-sided). Lysates of primary bone marrow ECs from WT or *Stk11*^ec−/−^ mice were analyzed by western blotting for Scf, Lkb1, and β-Actin (**b**, lower), and by PCR for *Scf* (**c**, upper). The serum Scf level from WT or *Stk11*^ec−/−^ mice was detected by ELISA (**c**, lower; 12-weeks-old, mixed-gender, WT, *n* = 14; *Stk11*^ec−/−^, *n* = 13). *P < 0.001 versus WT by Student’s *t*-test (two-sided). The blots are representative results of three independent experiments. **d** Bone marrow cells (1 × 10^6^ cells/per well) isolated from WT or *Stk11*^ec−/−^ mice were cultured with or without c-Kit inhibitor ISCK03 (1 µM) for 6 days to generate DCs. Primary DCs were harvested from each dish by collecting non and loosely adherent cells and stained for CD11c, detected by flow cytometry. DCs were defined as CD11c^+^ cells. The colonies (red head) and megakaryocytes (green head) were stained with Giemsa. Scale bar: 1000 μm. Bar graph summarizes the numbers of DCs and megakaryocytes per cm^2^ in culture. *P < 0.001 versus WT + vehicle by Student’s *t*-test (two-sided). **e** Bone marrow cells (1 × 10^6^ cells/per well) isolated from WT or *Stk11*^ec−/−^ mice were cultured with or without recombinant murine stem cell factor (rmScf, 50 ng/mL) for 6 days to generate DCs. Primary DCs were harvested from each dish by collecting nonadherent and loosely adherent cells and stained for CD11c, detected by flow cytometry. DCs were defined as CD11c^+^ cells. The colonies ^(^Red head) and megakaryocytes (green head) were stained with Giemsa. Scale bar: 1000 μm. Bar graph summarizes the numbers of DCs and megakaryocytes per cm^2^ in culture. *P < 0.001 versus WT + Vehicle and ^†^*P* < 0.001 versus *Stk11*^ec−/−^ + vehicle by Student’s *t*-test (two-sided). **f** Flow cytometry analysis of spleen/lymph node cells isolated from WT or *Stk11*^ec−/−^ mice treated with either AAV-acGFP or AAV-sScf and stained for CD11c and MHC II or CD11c and B220. Bar graph summarizes frequency of spleen and lymph node cDCs and pDCs (12-weeks-old, mixed-gender, *n* = 5–6 each group). *P < 0.001 versus WT and ^†^*P* < 0.001 versus *Stk11*^ec−/−^ by nonparametric Mann–Whitney *U* test (two-sided). **g** Flow cytometry analysis of bone marrow cells isolated from WT or *Stk11*^ec−/−^ mice treated with either AAV-acGFP or AAV-sScf and stained for Lin, c-Kit, CD115, CX3CR1, and Flt3. Bar graph summarizes the ratio of MDP/CDP in bone marrow (12-weeks-old, mixed-gender, *n* = 5–7 each group). *P < 0.001 versus WT and ^†^*P* < 0.001 versus *Stk11*^ec−/−^ by nonparametric Mann–Whitney *U* test (two-sided).

Given the evidence of obstructed MDP-to-CDP differentiation in *Stk11*^ec−/−^ mice, but not *Stk11*^dc−/−^ mice, we next sought to examine the fate and distribution of MDP and CDP in mice bone marrow. We used green fluorescent protein (GFP) *Cx3cr1* knock-in mice (hereafter designated as *Cx3cr1*^*GFP*^). In these mice, MDP and their progeny cells are irreversibly labeled with GFP. Interestingly, the distribution of *Cx3cr1*-GFP^+^ c-Kit^+^ cells in bone marrow indicating MDP/CDP were more common in the bone marrow perivascular area but not directly in contact with vessels (Fig. 4a). Approximately 85% of MDP/CDP were 20 μm or far away from a sinusoidal blood vessel (Supplementary Movie 1), suggesting a cell-detached means of communication between bone marrow ECs and DC precursors.

### Soluble Scf is a key extrinsic perivascular niche cytokine for DC differentiation in mouse bone marrow

Deeper analysis of the most differentially expressed genes between primary ECs isolated from WT and *Stk11*^ec−/−^ mice highlighted extracellular protein binding as the most changed pathway based on the number of changed genes and statistical significance (Supplementary Fig. 9a). Among the factors involved in extracellular protein binding, we found that *Stk11* deletion in ECs reduced soluble Scf, whereas increased membrane-bound Scf mRNA and protein levels (Fig. 4b). *Stk11*^ec−/−^ mice showed a 50% reduction in serum soluble Scf level, compared with littermate control mice (Fig. 4c). Additionally, Jak2 and Akt phosphorylation decreased in MDP, but not terminal differentiated DC (classical DC and plasmacytoid DC) in *Stk11*^ec−/−^ mice compared with their littermate controls (Supplementary Fig. 9b, c).

To explore whether Scf is required for MDP differentiation into CDP, unfractionated bone marrow cells from *Stk11*^ec−/−^ mice and littermate control mice were treated with or without ISCK03, a cell-permeable inhibitor of the Scf receptor c-Kit. In the presence of ISCK03 in culture, both DC production and colony formation reduced sharply in bone marrow cells of littermate control mice, associated with an increased number of megakaryocytes (Fig. 4d). Interestingly, ISCK03 did not further impair DC differentiation in the *Stk11*^ec−/−^ mouse bone marrow cultures compared with vehicle (Fig. 4d).

Next, we cultured the unfractionated bone marrow cells from *Stk11*^ec−/−^ mice and littermate control mice in the presence or absence of recombinant murine soluble Scf (rmScf). Relative to littermate control mice, *Stk11*^ec−/−^ mouse bone marrow cells showed a 60% reduction in the percentage of colonies and DC production without rmScf supplementation (Fig. 4e). Addition of rmScf markedly increased colony number and DC production in littermate control mice and *Stk11*^ec−/−^ mouse bone marrow cultures (Fig. 4e), suggesting a compensatory function of rmScf in DC differentiation in *Stk11*^ec−/−^ mouse bone marrow cells. Similarly, bone marrow cells from the *Stk11*^ec−/−^ mice showed a ten-fold induction of megakaryocytes compared with the unfractionated bone marrow cells of WT mice, whereas in the presence of rmScf, there is negligible megakaryocyte production in either the littermate control mice or the *Stk11*^ec−/−^ bone marrow in culture (Fig. 4e).

To further distinguish whether DC shortage in *Stk11*^ec−/−^ mice is due to hematopoietic cells or defective niche cells, *Stk11*^ec−/−^ mice were transplanted with bone marrows from WT or *Stk11*^ec−/−^ mice, and vice versa. As shown in Supplementary Fig. 9d, there are no difference in the number of classical DCs and plasmacytoid DCs between WT mice receiving WT or *Stk11*^ec−/−^ mice bone marrows. In contrast, *Stk11*^ec−/−^ mice receiving WT mice bone marrow still showed lower number of classical DCs and plasmacytoid DCs than those WT mice (Supplementary Fig. 9d). Consistent with this observation, MDPs isolated from either WT or *Stk11*^ec−/−^ mouse bone marrow showed no difference on progenitor cell maintenance when cultured on standard AFT024 feeder cells (Supplementary Fig. 9e). Taken together, those results indicated an extrinsic perivascular niche effect of soluble Scf on DC differentiation.

### Administration of soluble Scf restores MDP-to-CDP differentiation and DC lineage in *Stk11*^ec−/−^ mice in vivo

Next, we generated adeno-associated virus (AAV) encoding soluble Scf (AAV-sScf) or aequorea coerulescens GFP (AAV-acGFP) to systematically identify the function of soluble Scf in DC differentiation in vivo. Relative to *Stk11*^ec−/−^ mice treated with AAV-acGFP, the frequency of classical DCs and plasmacytoid DCs significantly increased in *Stk11*^ec−/−^ mice treated with AAV-sScf (Fig. 4f). Consistently, MDP-to-CDP differentiation was restored in the bone marrow of *Stk11*^ec−/−^ mice treated with AAV-sScf (Fig. 4g). Moreover, spleen size and megakaryocyte infiltration decreased with AAV-sScf treatment in *Stk11*^ec−/−^ mice, as compared with mice treated with AAV-acGFP (Supplementary Fig. 10b, c).

### Lkb1-dependent phosphorylation of Ptbp1 inhibits *Scf* alternative splicing

**Fig. 5:**
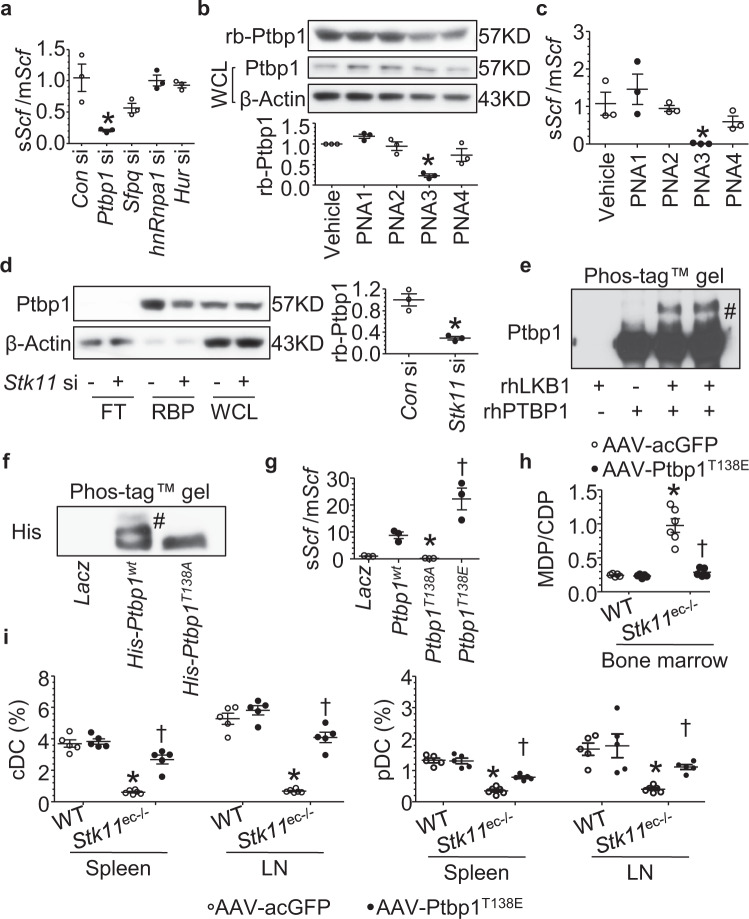
Lkb1-dependent Ptbp1 phosphorylation promotes *Scf* exon 6 retention to induce the formation of soluble stem cell factor (sScf) and drives DC differentiation. **a** Reverse transcription-polymerase chain reaction (RT-PCR) analysis of soluble *Scf* (including exon 6) to membrane-bound *Scf* (lacks exon 6) in control or target-gene-silenced primary mouse bone marrow ECs. *P = 0.02 versus *Control* si by nonparametric Mann–Whitney *U* test (two-sided). **b** RNA-binding protein was pull down using oligo-dT beads in indicated conditions and subjected to western blotting of Ptbp1 binding. *P = 0.02 versus vehicle by nonparametric Mann–Whitney *U* test (two-sided). **c** RT-PCR analysis of soluble *Scf*/membrane-bound *Scf* ratio in vehicle or peptide nucleic acid (PNA)-treated bone marrow ECs. *P = 0.03 versus vehicle by nonparametric Mann–Whitney *U* test (two-sided). **d** RNA-binding protein (RBP) was pulled down using oligo-dT beads in WT (vehicle-treated) or PNA competition assay and subjected to western blotting of Ptbp1 binding. FT, flow-through; rb-Ptbp1, RNA binding Ptbp1; WCL, whole-cell lysate. *P = 0.004 versus *Control* si by nonparametric Mann–Whitney *U* test (two-sided). **e** Proteins in in vitro kinase assay reaction mixture were separated on a Phos-tag^TM^ gel and analyzed by western blotting with the anti-Ptbp1 antibody. # marked the phosphorylated band. Blots are representative of three independent experiments. **f** Endothelial cells (ECs) were transfected with His-*Ptbp1*^*wt*^ and mutant plasmid and whole-cell lysates were analyzed by western blotting for anti-His after separation in Phos-tag^TM^ gel. # marked the phosphorylated band. Blots are representative of three independent experiments. **g** PCR analysis was carried out to detect soluble *Scf*/membrane-bound *Scf* ratio in BAECs transfected with *Ptbp1*^*wt*^ and mutant plasmid. *P = 0.003; ^†^*P* = 0.007 by nonparametric Mann–Whitney *U* test (two-sided). **h** Flow cytometry analysis of bone marrow cells isolated from WT or *Stk11*^ec−/−^ mice treated with either AAV-acGFP or AAV-Ptbp1^T138*E*^ and stained for Lin, c-Kit, CD115, CX3CR1, and Flt3. Bar graph summarizes the ratio of MDP/CDP in bone marrow (12-weeks-old, mixed-gender, *n* = 5–6 each group). *P < 0.001 versus WT and ^†^*P* < 0.001 versus *Stk11*^ec−/−^ by nonparametric Mann–Whitney *U* test (two-sided). **i** Flow cytometry analysis of spleen/lymph node cells isolated from WT or *Stk11*^ec−/−^ mice treated with either AAV-acGFP or AAV-Ptbp1^T138E^ and stained for CD11c and MHC II or CD11c and B220. Bar graph summarizes the frequency of cDCs and pDCs in spleen, and lymph node (12-weeks-old, mixed-gender, *n* = 5–6 each group). *P < 0.001 versus WT and ^†^*P* < 0.001 versus *Stk11*^ec−/−^ by nonparametric Mann–Whitney *U* test (two-sided).

Given the evidence of decreased soluble Scf and increased membrane-bound Scf mRNA and protein in *Stk11*-deleted ECs, we next thought to identify the splicing element that directly regulates *Scf* alternative splicing. The particular high degree of sequence conservation of *Scf* exon 6 during evolution raises the hypothesis that exon 6 might be the target of a sequence-specific RNA-binding protein^21^, and bioinformatic analysis revealed several candidates (Supplementary Fig. 11a). Among these, only polypyrimidine tract binding protein 1 (*Ptbp1*) knockdown enhanced *Scf* alternative splicing (Fig. 5a). We next designed and generated four different peptides nucleic acid (PNA) isoforms based on the *Scf* exon 6 sequence to inhibit RNA-binding protein binding (Supplementary Fig. 11b). Among these, only PNA3 which prohibited Ptbp1 binding to the mRNA (Fig. 5b), dramatically reduced the soluble Scf/membrane-bound Scf ratio (Fig. 5c). Interestingly, an efficient knockdown of *Stk11* inhibited Ptbp1 binding to mRNA (Fig. 5d), suggesting that Lkb1 may promote *Scf* alternative splicing through Ptbp1. We next tried to determine how Lkb1 regulates Ptbp1 binding. Because phosphorylation of Ptbp1 dramatically decreases upon *Stk11* deficiency (Supplementary Fig. 11c, d), Ptbp1-driven *Scf* alternative splicing is likely due to Lkb1 phosphorylation. To this end, we performed in vitro kinase assay and found that Lkb1 directly phosphorylates Ptbp1 (Fig. 5e). Additionally, *Stk11* deletion in ECs decreased Ptbp1-Lkb1 binding (Supplementary Fig. 11e). Computer alignment with an optimal Lkb1 substrate motif showed that Ptbp1 residue Thr138 is a potential Lkb1 phosphorylation target site (Supplementary Fig. 11f). Therefore, we generated two site-directed mutant constructs of *Ptbp1* (*Ptbp1*^*T138A*^ and *Ptbp1*^*T138E*^). Phosphorylation of Ptbp1 at Thr138 was abolished in ECs expressing *Ptbp1*^*T138A*^ (Fig. 5f), implying that Thr138 is the target of Lkb1-mediated phosphorylation. We further determined whether Ptbp1 Thr138 phosphorylation was required for the alternative splicing of *Scf*. To this end, we found that *Ptbp1*^*T138A*^ mutation decreased the soluble Scf/membrane-bound Scf ratio, whereas *Ptbp1*^*T138E*^ mutation increased soluble Scf/membrane-bound Scf ratio (Fig. 5g) and increased PTBP1-*Scf* (exon 6) binding (Supplementary Fig. 11g). Deeper analysis of the most differentially expressed genes among ECs overexpressed *Ptbp1*^*T138A*^, *Ptbp1*^*T138E*^, and *Ptbp1*^*wt*^ mutant further highlighted protein binding and adheshion as the most changed pathyway based on the number of changed genes and statistical significance (Supplementary Fig. 11h). Notably, the protein binding and adheshion pathway also stood out in the RNAseq data set with *Stk11* deficiency (Supplementary Fig. 9a). Taken together, these findings imply that Lkb1 phosphorylated Ptbp1 at Thr138, initiating Ptbp1 binding to *Scf* exon 6 and alternative splicing.

### Administration of *Ptbp1*^*T138E*^ restores MDP-to-CDP differentiation and DC lineage in *Stk11*^ec−/−^ mice in vivo

Given the evidence that phosphorylation of Ptbp1 Thr138 promotes soluble Scf generation, we next sought to identify the function of Ptbp1 Thr138 phosphorylation in DC lineage differentiation in vivo. To this end, we generated recombinant AAV encoding *Ptbp1*^*T138E*^ (AAV-Ptbp1^T138E^) or ac*GFP* (AAV-acGFP). SCF expression and MDP-to-CDP differentiation was restored in the bone marrow of *Stk11*^ec−/−^ mice treated with AAV-Ptbp1^T138E^ (Supplementary Fig. 10a, Fig. 5h). The frequencies of classical DC and plasmacytoid DC significantly increased in *Stk11*^ec−/−^ mice treated with AAV-Ptbp1^T138E^ (Fig. 5i). Consistent with this observation, spleen size and megakaryocyte infiltration decreased with AAV-Ptbp1^T138E^ treatment in *Stk11*^ec−/−^ mice compared with AAV-acGFP transduction (Supplementary Fig. 10b, c).

## Discussion

In this study, we showed that *Stk11*-guided alternative splicing of *Scf* in bone marrow ECs is strictly and non-cell-autonomously required for MDP differentiation into CDP. Mechanistically, endothelial Lkb1 (encoded by *Stk11*) regulates *Scf* alternative splicing through phosphorylation of Ptbp1 residue threonine 138. Phosphorylated Ptbp1 binds to *Scf* pre-mRNA to promote *Scf* exon 6 retention, thus suppressing *Scf* alternative splicing in favor of soluble Scf. The reduced level of soluble Scf in *Stk11*^ec−/−^ mouse bone marrow disrupted MDP-to-CDP differentiation, resulting in inadequate numbers of classical DCs and plasmacytoid DCs in peripheral lymphoid organs.

The most important finding from this study is that we have for the first-time presented evidence that *Stk11* in bone marrow ECs plays a key role in DC differentiation, and thus regulates immune homeostatic equilibrium. *Stk11*^ec−/−^ mice showed a disruption of MDP-to-CDP differentiation and reduced DC number. Consequently, inadequate DCs led to defective or dysregulated tumor immune surveillance, manifested as spontaneous tumor development in *Stk11*^ec−/−^ mice. Furthermore, the reduced number of DCs led to aberrant lymphocyte activation, inducing tissue inflammation, and disorganized immune activation. This also resulted in compensatory hematopoiesis, as myeloid proliferation disorder and splenomegaly in the *Stk11*^ec−/−^ mice. These phenomena (high premature death rate, spontaneous tumor development, aberrant lymphocyte activation, and myeloid proliferation) in *Stk11*^ec−/−^ mice are extremely similar to the clinical DC deficiency syndrome^46,47^. Some patients with DC deficiency syndromes become seriously ill and die in their early 30s. Those who survive show a high incidence of solid tumors, autoimmune disease, and myeloproliferation^46,47^. Notably, there is no clear identified pathogen or cause for the syndromes, except two mutations, GATA-binding factor 2 (GATA2) and interferon regulatory factor 8 (IRF8)^46,47^. Our findings constitute an important example of how the loss of tumor suppressor in bone marrow ECs or niche cells drives specific DC precursor cell differentiation and spontaneous tumor development. We also suggest that the *Stk11*-Ptbp1-Scf axis may be a potential new therapeutic avenues for DC deficient syndrome for which there are no treatment options right now.

Another significant finding of this study is that disrupted MDP-to-CDP differentiation after *Stk11* deletion in ECs appears to depend on soluble Scf, as shown by the evidence that *Stk11* deletion in ECs decreased soluble Scf and increased membrane-bound Scf. The addition of soluble Scf restored DC differentiation and the MDPs/CDPs ratio both in vivo and in vitro. Scf has long been suggested to be required for HSC survival, as HSCs are depleted in *Tie2*-Cre-mediated EC-*Scf*-deleted mice (*Scf*^*fl/-*^*Tie2*^cre^ mice)^17^. In our case, disrupted MDP-to-CDP differentiation in *Stk11*^ec−/−^ mice seems independent of HSC and other lineage-restricted HPC, as HSC, MPP, LMPP, CLP, CMP, GMP, and other restricted-lineage cells were unaffected. Membrane-bound Scf is not reduced (actually increased) in *Stk11-*deleted ECs, while membrane-bound Scf is more important for HSC maintenance and self-renewal^22,23^, whereas soluble Scf is more important for the normal development of restricted lineages, e.g., the DC lineage in this case. The different distribution of membrane-bound and soluble Scf raised the possibility that physical distance within the niche between HSC/HPC and ECs may affect the regulation and fate of stem cells and progenitor cells. Deep imaging of mouse bone marrow further supports this hypothesis, as shown by the fact that MDP/CDP reside mainly in perivascular niches adjacent to, but not in contact with, bone marrow ECs.

In the current study, we present evidence that Lkb1 in ECs regulates membrane-bound Scf/soluble Scf expression through Ptbp1-phosphorylation-mediated *Scf* alternative mRNA splicing. It has been reported that an acidic microenvironment might be responsible for the switch of *Scf* mRNA splicing in favor of membrane-bound Scf^21^. But it is unclear how this alternative mRNA splicing happens. In this study, using four different sequence-specific PNAs to compete with the target *Scf* exon 6 sequence, we found that Ptbp1 is the key to *Scf* exon 6 skipping and alternative splicing. Lkb1-dependent phosphorylation of Ptbp1 at Thr138 is required for Ptbp1 binding to *Scf* pre-mRNA exon 6. The binding thus switches *Scf* mRNA alternative splicing in favor of soluble Scf.

In sum, our data demonstrates that *Stk11* in bone marrrow ECs is strictly and non-cell-autonomously required for DC lineage and MDP-to-CDP differentiation through Ptbp1-phosphorylation-dependent *Scf* alternative splicing. These newly recognized niche features of DCs open up the possibility of expanding or reducing DC numbers by lineage-restricted cytokines or pathways in vivo, and provide new therapeutic opportunities for DC deficiency syndromes.

## Methods

### Mice

*Stk11*^fl/fl^ (#014143), *Cdh5*^Cre^ (#006137), *Cx3cr1*^Cre^ (#025524), *Cx3cr1*^GFP^ (#005582), *Gt(ROSA)26Sor*^tm1(EYFP)Cos^/J (#006148) mice were obtained from the Jackson Laboratory. VECad-Cre^ERT2^ mice were provided by Dr. Xin Zhang (University of Oklahoma Health Science Center). *Stk11*^fl/fl^, *Cdh5*^Cre^, and *Stk11*^fl/fl^*Cdh5*^Cre^ (EC-specific *Stk11-*deleted, *Stk11*^ec−/−^) mice were generated as previously described^38^. *Stk11*^fl/fl^*ROSA*^EYFP^*Cdh5*^cre^ mice were generated by crossing breeding *Stk11*^fl/fl^*Cdh5*^Cre^ mice with *Gt(ROSA)26Sor*^tm1(EYFP)Cos^/J reporter mice. *Stk11*^fl/fl^*Cx3cr1*^Cre^ (DC-specific *Stk11-*deleted, *Stk11*^dc−/−^) were generated by crossing breeding *Stk11*^fl/fl^ mice with *Cx3cr1*^Cre^ mice. *Stk11*^fl/fl^VECad-Cre^ERT2^ mice were generated by crossing breeding *Stk11*^fl/fl^ mice with VECad-Cre^ERT2^ mice. Tamoxifen citrate (Sigma, # PHR2706) was intraperitoneally (i.p.) injected at 75 mg/kg body weight with corn oil (Sigma, #C8267) to either *Stk11*^fl/fl^VECad-Cre^ERT2^ mice or littermate control mice at a concentration of 20 mg/mL every 24 h for a total of 5 consecutive days. Primers for genotyping are listed in Supplementary Table. 4. Mice were housed in a controlled environment (20 ± 2 °C, 12-h/12-h light/dark cycle) in specific pathogen-free (SPF) animal facility. Experimental mice were co-housed with control mice. When euthanized, carbon dioxide inhalation chamber followed by cervical dislocation was performed. All animal protocols were approved by the Georgia State University Committee on the Use and Care of Animals (IACUC number: A18053).

### Two-photon deep imaging of bone marrow

Freshly dissected tibias from 8 to 12-week-old mice were fixed for 6–8 h in 4% paraformaldehyde in phosphate-buffered saline (PBS) at 4 °C. Tibias were sectioned perpendicular to the long axis into 300-μm thick sections using a Leica VT100 S vibratome. Sections were blocked in a staining solution containing anti-CD16/32 mouse Fc-blocking antibody overnight on a rotator at room temperature. The staining solution contained 10% dimethyl sulfoxide (Sigma, #276855), 0.5% IgePal630 (Sigma, # I3021), and 5% donkey serum (Jackson Immuno, # 017-000-121) in PBS. Bone sections were stained with green fluorescent protein (GFP), c-Kit, and laminin antibodies for 3 days, washed overnight in several changes of PBS then incubated for 3 days in a staining solution containing secondary antibodies. Antibody dilutions are listed in Supplementary Table. 7. The stained bone sections were dehydrated in a methanol series and cleared in benzyl alcohol: benzyl benzoate 1:2 mix (BABB clearing). 3D microscopy of the bone marrow was performed on a Confocal/Multiphoton Zeiss Leica SP8 resonant scanning confocal microscope with two-photon excitation. Confocal tiled Z-stack images were rendered in 3D and analyzed using Imaris v. 7.7.1 software.

### Flow cytometry and isolation of hematopoietic progenitor cells

Mouse bone marrow cells were isolated by flushing the long bones with Ca^2+^- and Mg^2+^-free Hanks’ Balanced Salt Solution supplemented with 2% heat-inactivated bovine serum. Mouse spleen, thymus, or lymph node cells were collected by crushing the fresh tissue between two glass slides followed by trituration. Blood was collected by cardiac puncture before euthanasia and mixed with 50 mM EDTA to prevent clotting. Cells were dissociated to a single-cell suspension by passing through a 25G needle several times and filtered through a 70-µm nylon mesh. Cells were counted using a Vi-Cell cell counter (Beckman Coulter). Anti-CD11c and anti-MHC class II antibodies were used to stain classical DCs. Anti-CD11c and anti-B220 were used to stain plasmacytoid DCs. Anti-Lin (CD3, CD19, CD49b, Ly6G), anti-B220, anti-F4/80, and anti-CD11b were used to stain macrophages. Anti-Lin (CD3, CD19, CD49b), anti-B220, anti-F4/80, and anti-CD45 were used to stain granulocytes. Anti-Lin (CD3, CD19, NK1.1, Ter119, B220), anti-CD11c, anti-MHC class II, anti-SIRPα and anti-Flt3 were used to stain pre-classical DCs. Anti-Lin (CD3, CD19, NK1.1, Ter119, B220), anti-c-Kit, anti-CD115, anti-CX3CR1, and anti-Flt3 were used to stain monocyte and dendritic cell progenitor (MDP). Anti-Lin (CD3, CD19, NK1.1, Ter119, B220), anti-c-Kit, anti-CD115, and anti-Flt3 were used to stain common DC progenitors. Antibody dilutions are listed in Supplementary Table. 7. All antibody labeling was performed at 4 °C for 20 min, followed by washing and centrifugation. Before flow cytometry analysis, cells were re-suspended in staining medium (BD, #554656) containing the viability dyes 7-aminoactinomycin D (7AAD) (BioLegend, #420404) to exclude dead cells during flow cytometry. Absolute counts by flow cytometry were performed using Precision Count Beads™ (BioLegend, #424902) according to the manufacturer’s instructions. Samples were run on LRS Fortessa or FACS Aria II flow cytometers. Data were analyzed using FACSDiva (BD Biosciences) or FlowJo (Tree Star) software. Representative FSC-SSC gates used in this study are shown in Supplementary Fig. 12.

### Flow cytometry sorting of bone marrow endothelial cells

Bone marrow endothelial cells (ECs) were stained by intravenous injection of 10 μg anti-VE-cadherin antibody (BD Biosciences, PE Rat Anti-Mouse CD144, #562243) 5-10 min before euthanizing the mice. Tibias and femurs were gently crushed using a mortar and pestle and then digested with DNase I (200 U/mL; ThermoFisher, # EN0521), Liberase^DL^ (250 mg/mL; Sigma, # 5401160001), type IV collagenase (1 mg/mL; ThermoFisher, # 17104019), and collagenase D (500 μg/mL; Sigma, # 11088858001) with agitation for 30 min at 37 °C. The cells were dissociated to a single-cell suspension by passing through a 25G needle several times and filtered with a 70-µm nylon mesh to generate a single-cell suspension. Cells were sorted in two successive rounds to ensure high purity using a FACS Aria II flow cytometer.

### Phos-tag^TM^ western blotting assay

For Phos-tag^TM^ western blot, 8% acrylamide gel was mixed with 50 μmol/L Phos-tag™ acrylamide (Fujifilm, #AAL-107) and 100 μmol/L ZnCl_2_ (Sigma, #229997). After sample separation on a Zn^2+^-Phos-tag SDS/PAGE gel, the gel was incubated with gentle agitation in transfer buffer supplemented with 1 mM EDTA, 3 times for 10 min each, followed by washing with transfer buffer without EDTA for another 10 min. Proteins were transferred to nitrocellulose membranes using a wet-tank method and analyzed by western blotting. Ptbp1, His, and β-actin signals were detected simultaneously using enhanced chemiluminescence (ECL) western blotting detection reagents (Thermo Fisher Scientific).

### In vitro kinase assays

Recombinant LKB1 kinase (100 ng) and/or PTBP1 (1ug) peptides were incubated in 50 mM Tris-HCL (pH 7.4) containing 1 mM DTT, 10 mM MgCl_2_ and 100 μM ATP for 30 min at 37 °C. The reaction mixture was supplemented with 20 µl 3× loading buffer (Fisher scientific, #NC9566545) to terminate the reaction, boiled for 10 min at 99 °C, and proteins were separated by Phos-tag^TM^ SDS-PAGE.

### RNA-protein pull-down assay

Human umbilical vein endothelial cells (HUVECs) were transfected with siRNA targeting *Stk11* (Santa Cruz, # sc-35817, 10 nM) and RNAiMAX (Invitrogen, # 13778030) at a ratio of 4:1 or treated with peptide nucleic acids (PNAs, 100 nM) (synthesis by PNA Bio). Cells were then harvested in lysis buffer (50 mM Tris-HCl, pH 7.4, 150 mM NaCl, 1 mM EDTA, 1% NP-40, with protease inhibitor cocktail (Sigma, # S8820) and RNase inhibitor (Sigam, # 3335399001) added immediately before use. To capture total mRNA-protein complexes, pre-washed oligo-dT magnetic beads (New England Biolabs, #S1419S) were added to cell lysates and incubated overnight at 4 °C. After washing the beads three times with washing buffer (50 mM Tris-HCl, pH 7.4, 150 mM NaCl), binding proteins were eluted with protein denaturing buffer (375 mM Tris-HCl, pH 6.8, 9% SDS, 50% glycerol, 9 % β-mercaptoethanol, and 0.03% bromophenol blue). The enrichment of RNA-binding proteins was analyzed by immunoblotting.

### Primary cell culture

Bone marrow cells were collected as described above. Cells (1 × 10^5^) were plated in Iscove’s Modified Dulbecco’s Medium (IMDM) (ATCC® 30-2005) supplemented with 20 ng/ml granulocyte-macrophage colony-stimulating factor (GM-CSF) (Peprotech, #315-03), and 1× penicillin/streptomycin in 6-well plates. Sorted MDP or common DC precursor cells were co-cultured with AFT024 (ATCC^®^ SCRC-1007^™^) stromal cells, plated in IMDM supplemented with 20 ng/ml GM-CSF and 1× penicillin/streptomycin in 6-well plates. Cells were incubated in a humidified atmosphere of 5% CO_2_ at 37 °C. Colonies were counted after staining with Giemsa (Sigma, #G5637) using an inverted microscope (Olympus, Tokyo, Japan).

### Histological analysis

Mouse tumor, femurs, spleen, and lymph node were dissected and cleaned, followed by fixation in 10% formalin and paraffin embedding. Femurs were decalcified in Decalcifying Solution-Lite (Sigma, #D0818). For morphological analyses, tissue sections (4-μm) were prepared and stained with hematoxylin and eosin (H&E). The histological features of the tissues were observed and images captured using a light microscope (Olympus, Tokyo, Japan).

### Immunofluorescence analysis

Snap-frozen mouse tissues were embedded in Optimal Cutting Temperature compound (OCT) and 8-μm sections were prepared. For immunofluorescent staining, tissue sections were incubated with antibodies against Von Willebrand factor (vWF) or Lkb1 and DAPI Staining Solution, followed by incubation with secondary antibody. Antibody dilutions are listed in Supplementary Table. 7. All sections were imaged with identical exposure settings, and the images were quantified using Image-Pro Plus 6.0 software. Quantification of the positive signal in regions of interest was performed as described above.

### Immunohistochemical staining

The immunohistochemical staining of paraffin-embedded tissue sections was completed as described above. Following heat-induced antigen retrieval and incubation with blocking buffer (DAKO), tissue sections were incubated with antibodies against Lkb1 or CD61. Antibody dilutions are listed in Supplementary Table. 7. The tissues were then incubated sequentially with labeled horseradish peroxidase (Dako Real EnVision-HRP, Rabbit-Mouse, #K4063), 3,3’ diaminobenzidine (DAB; Dako, #K4061), and hematoxylin (Sigma, St. Louis, MO). Positive and negative controls were included in each experiment. Tissue sections were mounted and visualized with an Olympus BX53 microscope. Images were captured and quantified with Image-Pro Plus 6.0 software, as described above.

### Enzyme-linked immunosorbent assay (ELISA)

Serum was obtained from peripheral blood after centrifugation. Scf levels in mouse serum or cell culture medium were determined using an ELISA kit (Abcam, #ab100740) according to the manufacturer’s instructions.

### Reverse transcription-polymerase chain reaction (RT-PCR) and quantitative PCR (qPCR)

Total mRNA was isolated using TRIzol reagent (Invitrogen, #15596018) and reverse-transcribed using the cDNA synthesis kit (Promega, #A5001) according to the manufacturer’s instructions. RT-PCR was performed using SYBR green on a C1000 Touch™ Thermal Cycler (Bio-Rad). PCR was performed using *AccuPower*® HotStart PCR PreMix from Bioneer on an S1000™ Thermal Cycler (Bio-Rad). Primers are listed in Supplementary Table 5. Sample RNA levels were normalized to that of *Gapdh*. Calculations were performed by the comparative method 2^−∆∆CT^.

### RNA sequencing and data processing

Total RNA was extracted from endothelial cells isolated from 8 to 12-week-old mice using TRIzol (Invitrogen, USA), followed by sequencing at LC Science (Houston, TX). High-quality reads were aligned to Mus GRCm38 genome by HISAT2. The expression levels of each gene were normalized to fragments per kilobase million (FKPM) reads. Paired differential gene expression analyses were performed using String Tie by addition of fold change >2 and *P*-value < 0.05. GO or KEGG enrichment analyses of differential genes were performed using Ballgown R package.

### Western blotting analysis

Cell and tissue extracts were separated by SDS-PAGE and transferred onto a nitrocellulose membrane. The membrane was then sequentially incubated with primary and secondary antibodies. Antibody dilutions are listed in Supplementary Table. 7. Protein signals were detected using enhanced chemiluminescence (ECL) western blotting detection reagents (Thermo Fisher Scientific).

### siRNA transfection

siRNAs targeting *Ptbp1* (sc-38280), *Sfpq* (sc-38304), *Hnrnpa1* (sc-270345), *Hur* (sc-35619), *Lkb*1 (sc-35816), and *control* siRNA (sc-37007) were obtained from Santa Cruz Biotechnology. The siRNA transfections were completed using the manufacturer’s protocol.

### Plasmid construction and transfection

*Ptbp1* cDNA clone (pQE30, Ptbp1 isoform1) was purchased from Addgene (#108591). Phosphorylation mutant T138A and phosphomimetic mutant T138E were generated using a QuikChange II site-directed mutagenesis kit (Agilent, # 200523) according to the manufacturer’s instructions. Primers used for point mutation are listed in Supplementary Table 6. All mutations were verified by DNA sequencing.

### Adeno-associated virus (AAV) constructs

The AAV delivery system was used to overexpress soluble *Scf*-aequorea coerulescens GFP (ac*GFP*) or *Ptbp1*^*T138E*^-ac*GFP* or ac*GFP* in mouse. We first amplified the CMV-acGFP cassette from pCMV6-AC-GFP (Origene, #PS100010) and cloned it into pAAV.CMV.PI.GFP.WPRE.bGH (Addgene, #105530) by replacing the CMV-chimeric intron-EGFP following digestion with NheI and HindIII. Then we cloned EC-specific promoter of *Flt-1* (2748 to 284) into this construct by replacing CMV promoter with NheI and SalI restriction enzymes according to a previous report^48^ to create the construct pAAV*-*Flt1-acGFP. The open reading frame (ORF) encoding soluble *Scf* or *Ptbp1*, without a stop codon, was cloned into pAAV*-*Flt1-acGFP following digestion with SalI and MluI to create pAAV*-*Flt1-sScf-acGFP or pAAV*-*Flt1-Ptbp1-acGFP, respectively. Vector pAAV*-*Flt1-acGFP served as a control. The AAV2 vector was generated by transfecting HEK293T cells with three plasmids (pAAV*-*Flt1-derived plasmid, AAV2 rep and cap genes plasmid, and the pAd helper plasmid). Titers of vector DNA were measured by quantitative PCR with vector-specific primers. Mice were injected with 100 μl virus containing 10^11^ AAV2 vector genomes via the tail vein.

### Statistical analyses

Data were analyzed with SPSS version 18.0 (IBM). All values are expressed as the mean ± standard error of the mean (SEM) unless otherwise stated. All experiments were performed at least three times unless otherwise stated.

When assessing the variance within two groups, the *F*-test was used. In the results describing more than two groups, the Brown-Forsythe test was applied to analyze the equal variance assumption. The Shapiro–Wilk test was performed to assess data normality. When analyzing the difference between the two groups, Student’s *t*-test with a two-tailed distribution was performed when the assumptions (equal variance and normal distribution) were satisfied. The nonparametric Mann–Whitney *U* test was applied to analyze the difference between two groups when the assumptions of equal variance and normally distributed data were not met. *P* < 0.05 was considered statistically significant unless otherwise stated.

### Reporting summary

Further information on research design is available in the Nature Research Reporting Summary linked to this article.

## Supplementary information

Supplementary Information

Description of Additional Supplementary Files

Supplementary Movie 1

Reporting Summary

## Data Availability

The RNAseq data is available in the Gene Expression Omnibus (GEO) database under accession code GSE184630 and GSE184631. All relevant data are provided as a Source data file, or available within the article and its Supplementary information files or upon request. Source data are provided with this paper.

## Source data

Source data
