# Supplementary material for: Endothelial cell-specific expression of serine/threonine kinase 11 modulates dendritic cell differentiation
Source: Nat Commun. 2022 Feb 3;13:648. doi: 10.1038/s41467-022-28316-6 (PMC8814147; doi:10.1038/s41467-022-28316-6)
Supplement: Supplementary file 1 — Supplementary Information [file 41467_2022_28316_MOESM1_ESM.pdf]

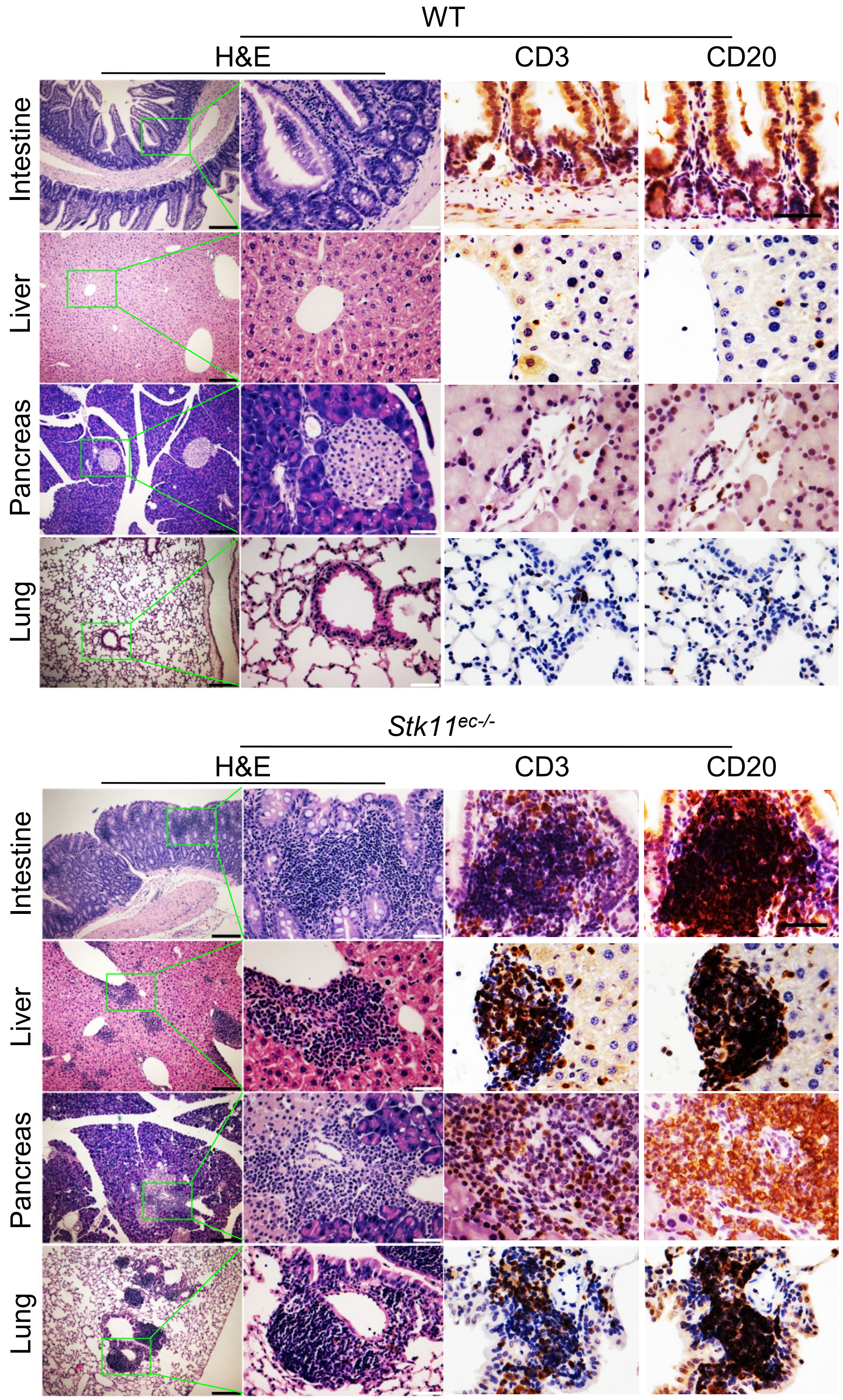

Supplementary Figure 1. *Stk11*$^{ec-/-}$ mice exhibit disorganized immune activation. Representative images of hematoxylin and eosin (H&E)/ CD3 /CD20-stained intestine, liver, pancreas, and lung sections. Scale bar: (Black) 200 μm, (White) 50 μm. Images are representative of three independent.

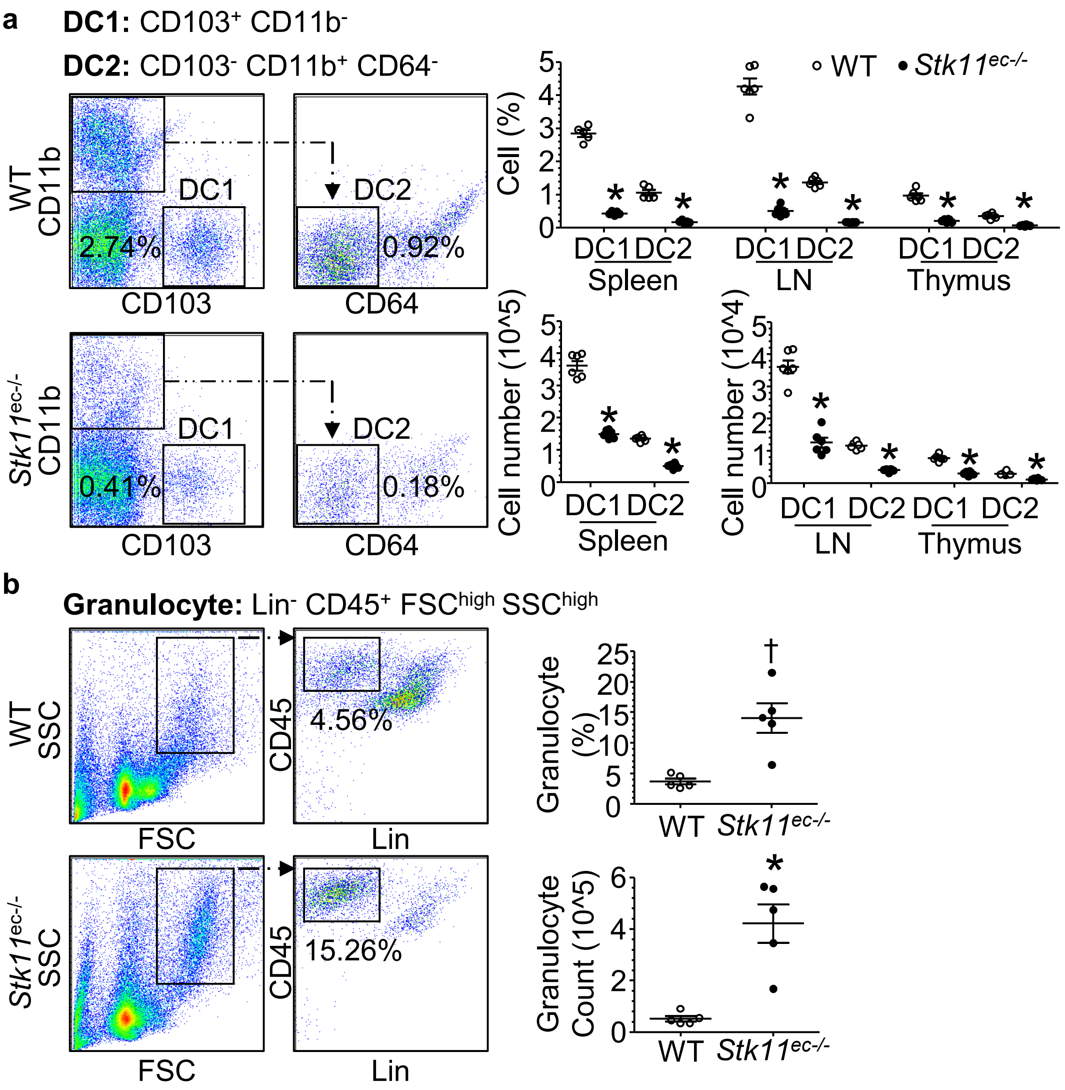

Supplementary Figure 2. ***Stk11*$^{ec-/-}$ mice exhibit a myeloid proliferative disorder with depressed DCs. (a)** Flow cytometry analysis of spleen, lymph node (LN), and thymus cells isolated from WT or *Stk11*$^{ec-/-}$ mice and stained for CD103, CD11b, and CD64. DC1 were defined as $CD103^{+}$ $CD11b^{-}$ and DC2 were defined as $CD103^{-}$ $CD11b^{+}$ $CD64^{-}$. Bar graph summarizes DC1 and DC2 numbers (12-weeks-old, mixed-gender, n=5-6 each group). $^{*}P<0.001$ versus WT by nonparametric Mann-Whitney U test (two-sided). **(b)** Flow cytometry analysis of spleen cells isolated from WT or *Stk11*$^{ec-/-}$ mice and stained for Lin (CD3, CD19, CD49b), and CD45. Granulocyte were defined as Lin^{-} $CD45^{+}$ $FSC^{high}SSC^{high}$. Bar graph summarizes granulocyte number in mouse spleen (12-weeks-old, mixed-gender, n=5 each group). $^{\dagger}P=0.001$; $^{*}P<0.001$ versus WT by nonparametric Mann-Whitney U test (two-sided).

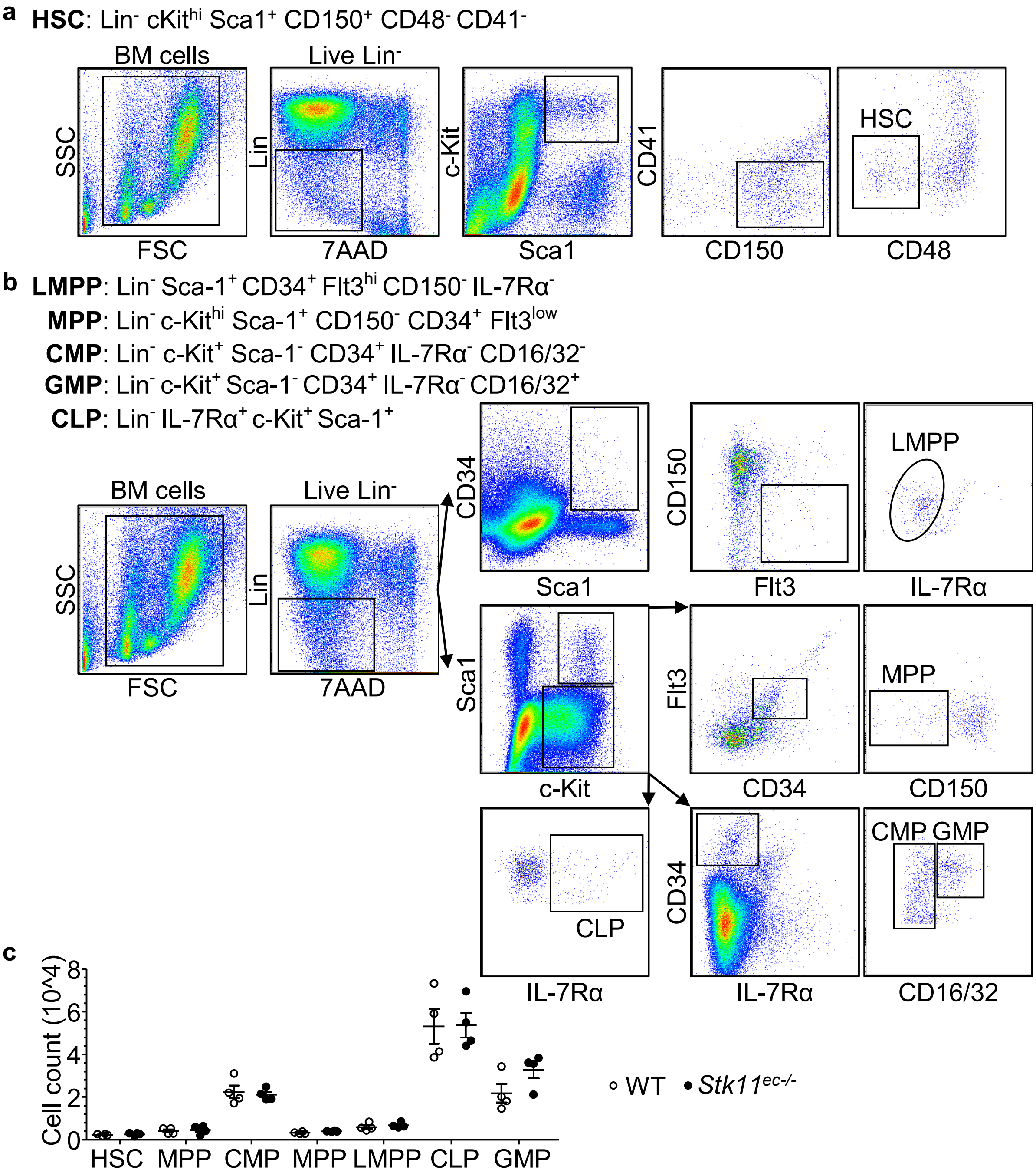

Supplementary Figure 3. Hematopoietic stem cells (HSCs) and hematopoietic progenitor cells (HPCs) count in bone marrow of WT or *Stk11*$^{ec-/-}$ mice. **(a)** Flow cytometric analysis of bone marrow cells isolated from WT or *Stk11*$^{ec-/-}$ mice and stained for Lin (CD3, CD14, CD16, CD19, CD20, CD56), c-Kit, Sca1, CD150, CD48, and CD41. Hematopoietic stem cells (HSCs) were defined as Lin^{-} c-Kit^{hi} Sca1^{+} CD150^{+} CD48^{-} CD41^{-}. **(b)** Flow cytometric analysis of bone marrow cells isolated from WT or *Stk11*$^{ec-/-}$ mice and stained for Lin (CD3, CD14, CD16, CD19, CD20, CD56), c-Kit, Sca1, CD34, Flt3, CD150, IL-7Rα and CD16/32. Lymphoid-primed multipotent progenitors (LMPPs) were defined as Lin^{-} Sca-1^{+} CD34^{+} Flt3^{hi} CD150^{-} IL-7Rα^{-}; Multipotent progenitors (MPPs) were defined as Lin^{-} c-Kit^{hi} Sca1^{+} CD150^{-} CD34^{+} Flt3^{low}; Common myeloid progenitors (CMP) were defined as Lin^{-} c-Kit^{+} Sca-1^{-} CD34^{+} IL-7Rα^{-} CD16/32^{-}; Granulocyte-monocyte progenitors (GMP) were defined as Lin^{-} c-Kit^{+} Sca-1^{-} CD34^{+} IL-7Rα^{-} CD16/32^{+}; Common lymphoid progenitors (CLP) were defined as Lin^{-} IL-7Rα^{+} c-Kit^{+} Sca-1^{+}. **(c)** Bar graph summarizes cell number of HSC, MPP, LMPP, CLP, CMP, and GMP (12-weeks-old, mixed-gender, n=4 each group).

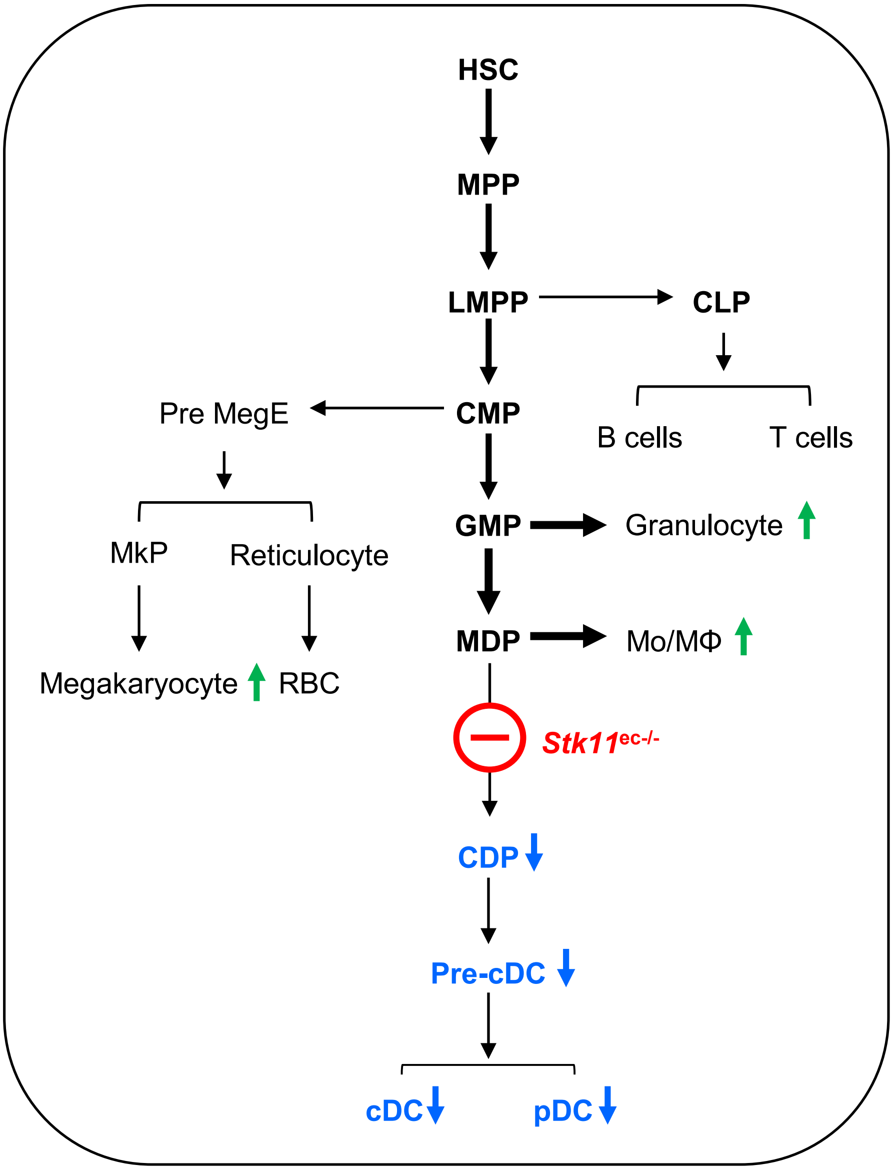

Supplementary Figure 4. Summarized information on hematopoietic-immune cells of *Stk11*$^{ec-/-}$ mice. HSC: hematopoietic stem cell; MPP: multipotent progenitor; LMPP: lymphoid-primed multipotent progenitor; CMP: common myeloid progenitor; CLP: common lymphoid progenitor; GMP: granulocyte-monocyte progenitor; MDP: macrophage and DC precursor; CDP: common DC precursor; pre-cDC: committed precursors of cDC; cDC: classical spleen DC; pDC: plasmacytoid DC; Pre MegE: precursor of megakaryocytes and erythrocytes; MkP: megakaryocyte precursor; RBC: red blood cell.

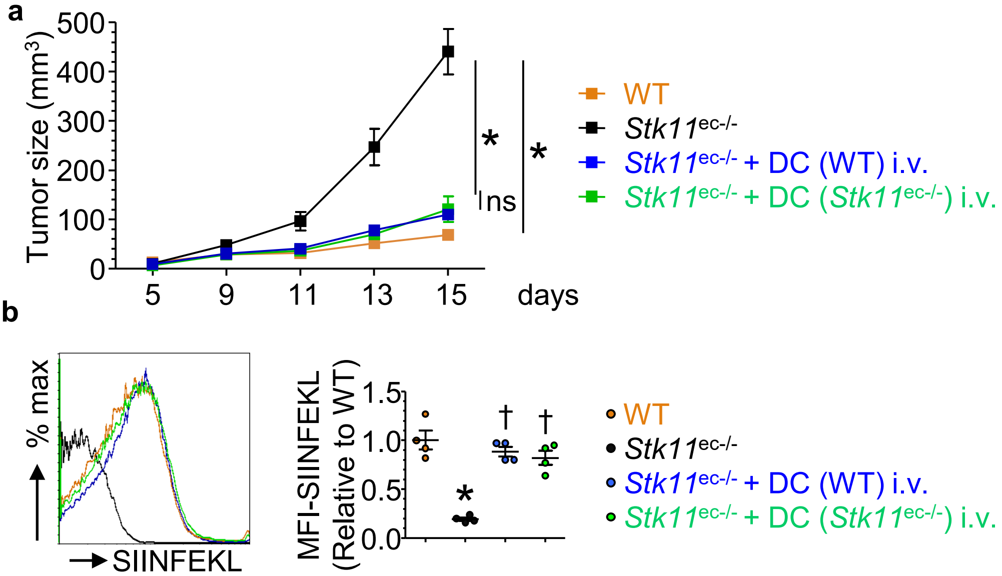

Supplementary Figure 5. Terminal differentiated DC function in *Stk11*$^{ec-/-}$ mice. **(a)** DCs (10^6) isolated from WT or *Stk11*$^{ec-/-}$ mice were tail vein injected into *Stk11*$^{ec-/-}$ mice 48 hours before subcutaneous injection of 10^5 B16-F10 mouse melanoma cells. Tumor size was monitored over time. $^*P<0.05$. **(b)** OVA epitope (SIINFEKL) complexes were examined in tumor-draining lymph nodes by flow cytometry. $^*P<0.001$ versus WT and $^\dagger P<0.05$ versus *Stk11*$^{ec-/-}$ by nonparametric Mann-Whitney U test (two-sided).

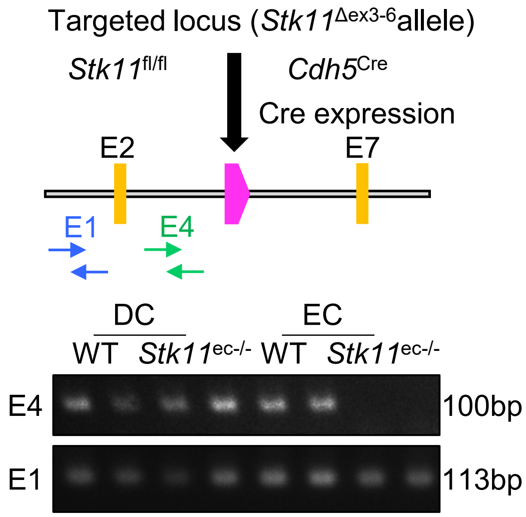

Supplementary Figure 6. Genotyping on ECs and DCs isolated from WT or *Stk11*$^{ec-/-}$ mice. Bone marrow ECs or spleen DCs were isolated from WT or *Stk11*$^{ec-/-}$ mice, subjecting for PCR using primers targeting *Stk11* exon 1 or exon 4. Blots are representative of three independent experiments.

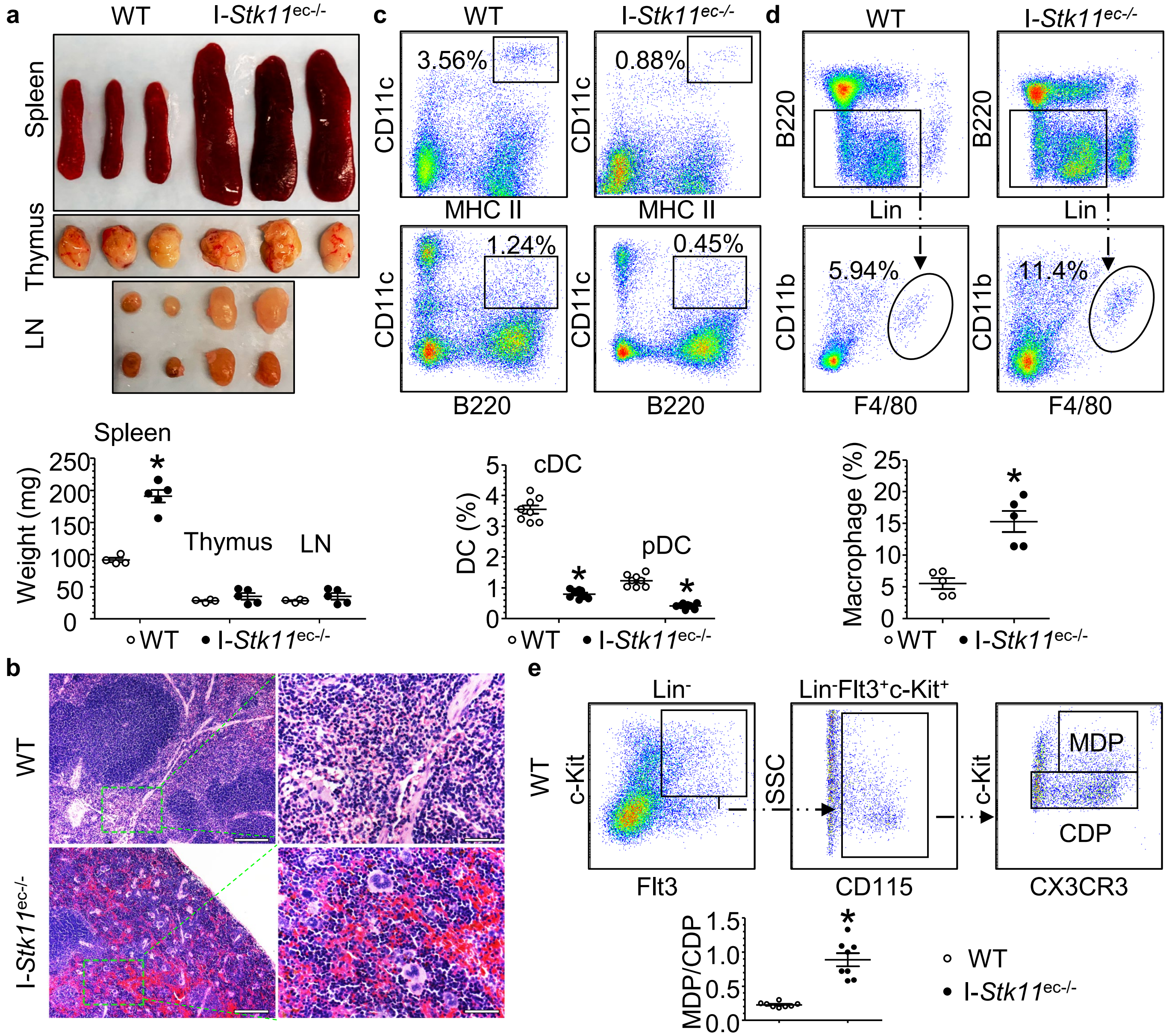

Supplementary Figure 7. Phenotypes of mice with EC-*Stk11* deletion at the age of 12 weeks. (a) Tamoxifen-inducible *Stk11*$^{ec-/-}$ mice were generated by cross-breeding *Stk11*$^{fl/fl}$ mice with VECad-Cre^{ERT2} mice and treated with tamoxifen at the age of 12 weeks. Representative images of spleen, thymus, and lymph node and quantification of organ weights of WT or inducible *Stk11*$^{ec-/-}$ mice (mixed-gender, n=4-5 each group). $^{*}P$<0.001 versus WT by nonparametric Mann-Whitney U test (two-sided). **(b)** Representative images of H&E-stained spleen of WT or inducible *Stk11*$^{ec-/-}$ mice. Scale bar: (left) 200 µm, (right) 50 µm. **(c-e)** Flow cytometric analysis of cells isolated from WT or inducible *Stk11*$^{ec-/-}$ mice. Bar graph summarizes frequency of spleen cDCs, pDCs, macrophages, and bone marrow MDP/CDP ratio (mixed-gender, n=5-8 each group). $^{*}P$<0.001 versus WT by nonparametric Mann-Whitney U test (two-sided).

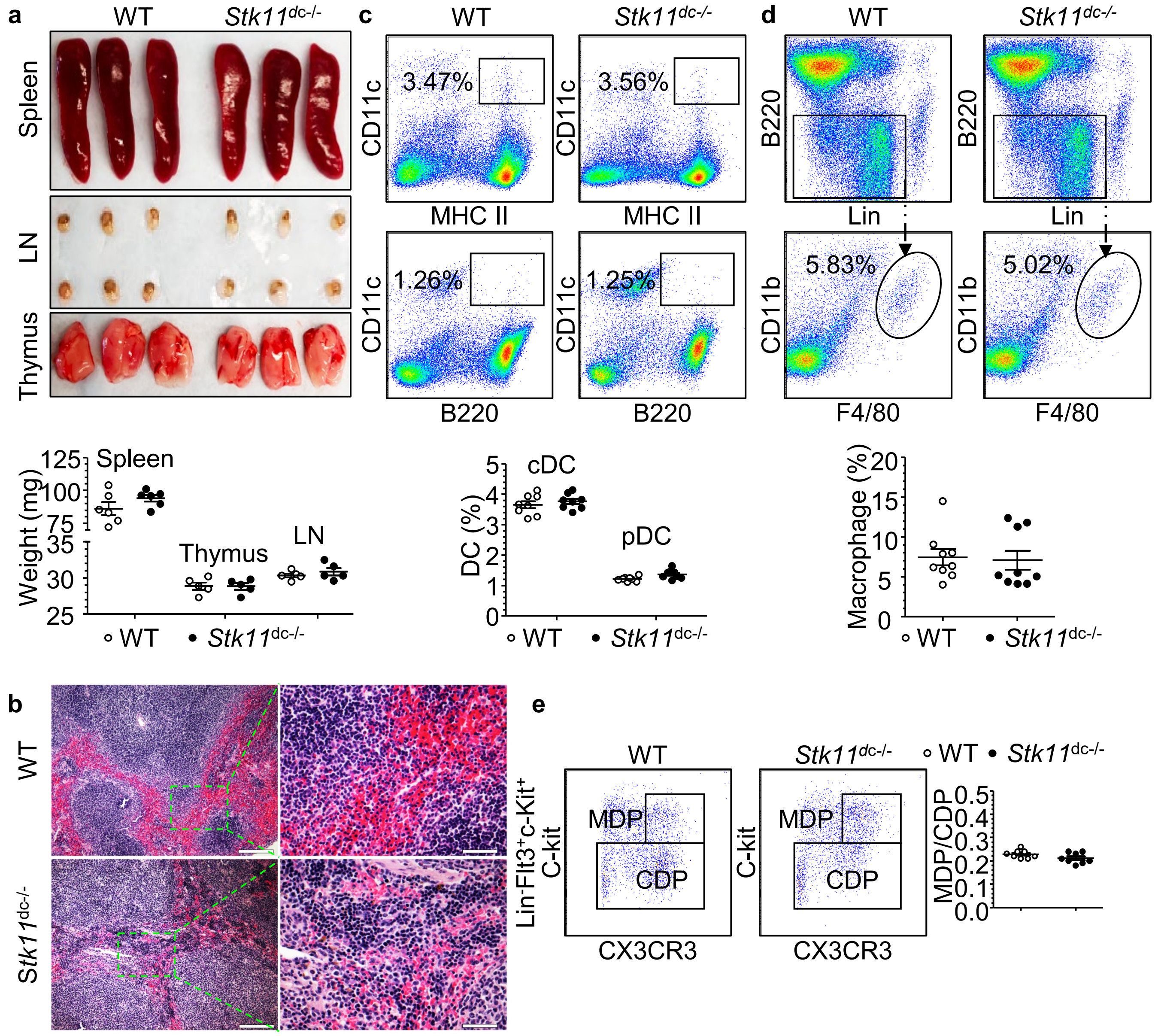

Supplementary Figure 8. DC-specific *Stk11* deleted mice (*Stk11*$^{dc-/-}$) show no difference on DC differentiation. (a) Representative spleen, lymph node (LN) and thymus images and quantification of spleen, LN and thymus weight of WT or *Stk11*$^{dc-/-}$ mice (12-weeks-old, mixed-gender, n=5-6 each group). **(b)** Representative images of H&E-stained spleen of WT or *Stk11*$^{dc-/-}$ mice. Scale bar: (left) 200 μm, (right) 50 μm. **(c-e)** Flow cytometry analysis of cells isolated from WT or *Stk11*$^{dc-/-}$ mice. Bar graph summarizes the frequency of spleen cDCs, pDCs, and macrophages, and bone marrow MDP/CDP ratio (12-weeks-old, mixed-gender, n=8-9 each group).

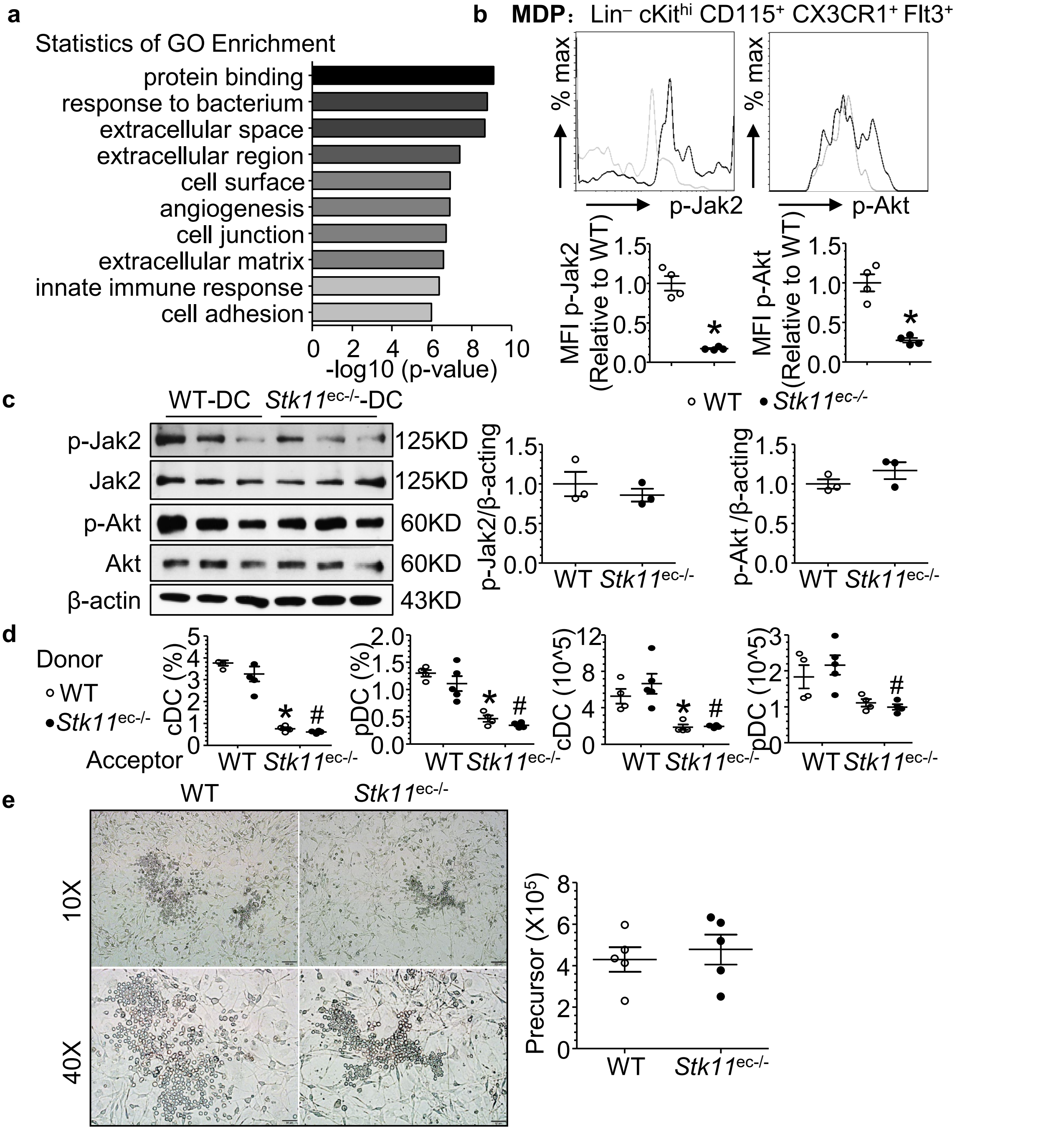

Supplementary Figure 9. Endothelial *Stk11* is non-cell-autonomous required for DC differentiation. (a) Gene ontology (GO) analysis of the identified differentially expressed genes between primary ECs from WT or *Stk11*ec-/- mice. The ten most significantly enriched pathways ($P < 0.05$ by Fisher's exact test) were shown. **(b)** Flow cytometry analysis of bone marrow cells isolated from WT or *Stk11*ec-/- mice and stained for Lin (CD3, CD19, Ter119, NK1.1, B220), CD11c, MHC II, SIRP-α, Flt3, and p-Jak2 (Tyr1007/1008) or p-Akt (Ser473). Bar graph summarizes mean fluorescence intensity (MFI) of Jak2 (Tyr1007/1008) or Akt (Ser473) in MDP from WT or *Stk11*ec-/- mice. $^{*}P<0.001$ versus WT by Student's *t*-test (two-sided). **(c)** Lysates of primary splenic DCs from WT or *Stk11*ec-/- mice were analyzed by western blotting for p-Jak2 (Tyr1007/1008), Jak2, p-Akt (Ser473), and Akt. **(d)** Irradiated WT or *Stk11*dc-/- mice were transplanted with bone marrows from WT or *Stk11*ec-/- mice. Flow cytometry analysis of spleen cells isolated from chimera mice and stained for CD11c and MHC II or CD11c and B220. Bar graph summarizes cell number of cDC and pDC in spleens (n=4-5 each group). $^{*}P<0.01$ versus WT and $^{\#}P<0.01$ versus *Stk11*ec-/- by nonparametric Mann-Whitney U test (two-sided). **(e)** Monocyte-dendritic cell progenitor cells (MDPs) were isolated from WT or *Stk11*ec-/- mice and co-cultured with AFT024 feeder cells in presence of GM-CSF (20 ng/ml) for 6 days. DC precursors were harvested from each dish by collecting loosely adherent cells. Scale bar: 100 µm.

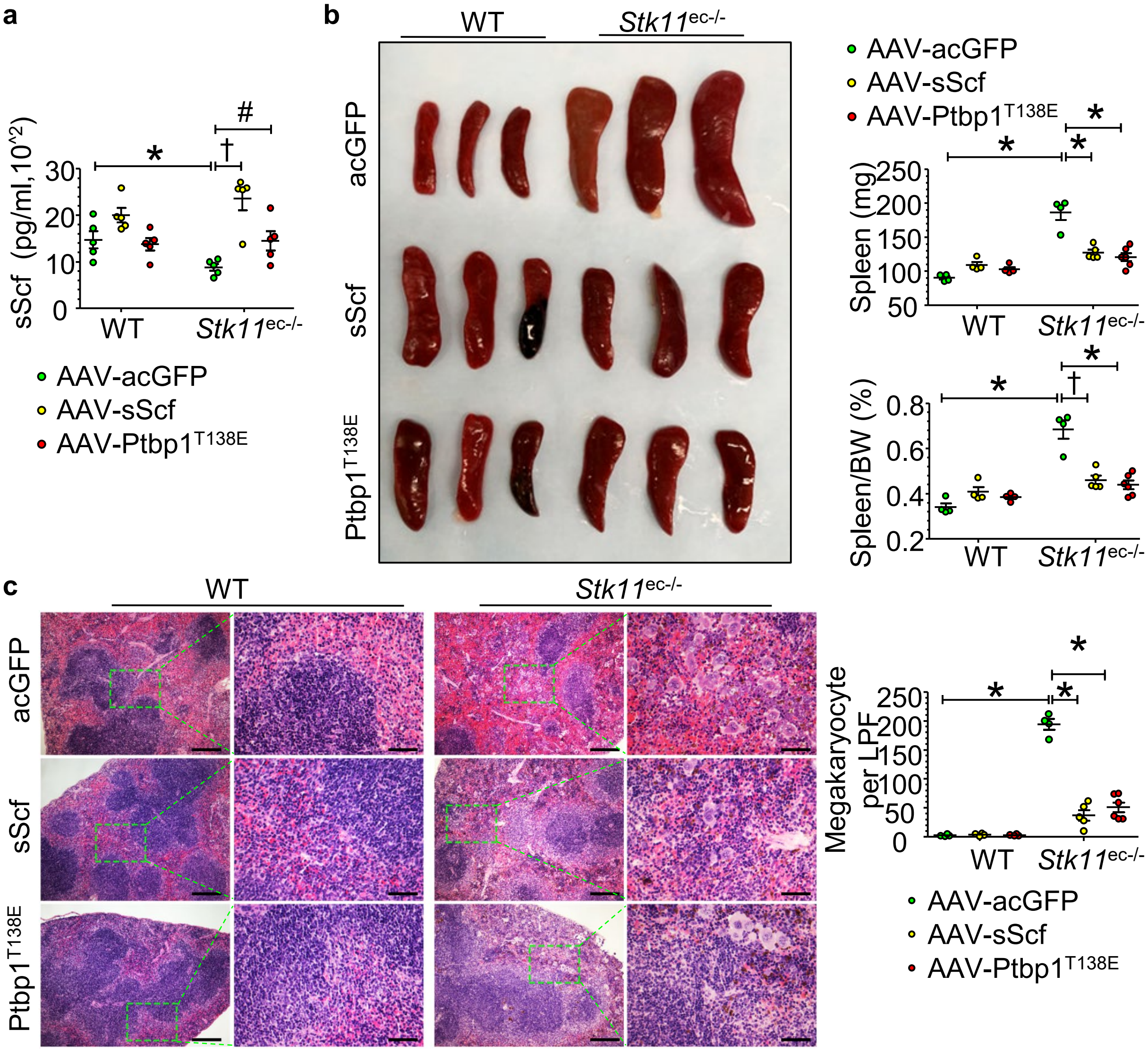

Supplementary Figure 10. Spleen size and structure of AAV-acGFP or AAV-sScf or AAV-Ptbp1^{T138E} treated mice. (a) ELISA analysis on serum Scf level from WT or *Stk11*$^{ec-/-}$ mice treated with AAV-acGFP or AAV-sScf or AAV-Ptbp1^{T138E}. $^{*}P$=0.02; $^{\dagger}P$=0.004; $^{\#}P$=0.03 by nonparametric Mann-Whitney U test (two-sided). **(b)** Representative images and quantification of spleen size and weight from WT or *Stk11*$^{ec-/-}$ mice transduced with either AAV-acGFP or AAV-sScf or AAV-Ptbp1^{T138E} (n=4-6 each group). $^{*}P$<0.001; $^{\dagger}P$=0.001 by nonparametric Mann-Whitney U test (two-sided). **(c)** Representative H&E-stained images of spleen of WT or *Stk11*$^{ec-/-}$ mice transduced with either AAV-acGFP, AAV-sScf, or AAV-Ptbp1^{T138E}. Scale bar: (left) 200 μm, (right) 50 μm. Bar graph summarizes the number of megakaryocytes per low power field (per LPF, 10× field, n=4-6 each group). $^{*}P$<0.001 by nonparametric Mann-Whitney U test (two-sided).

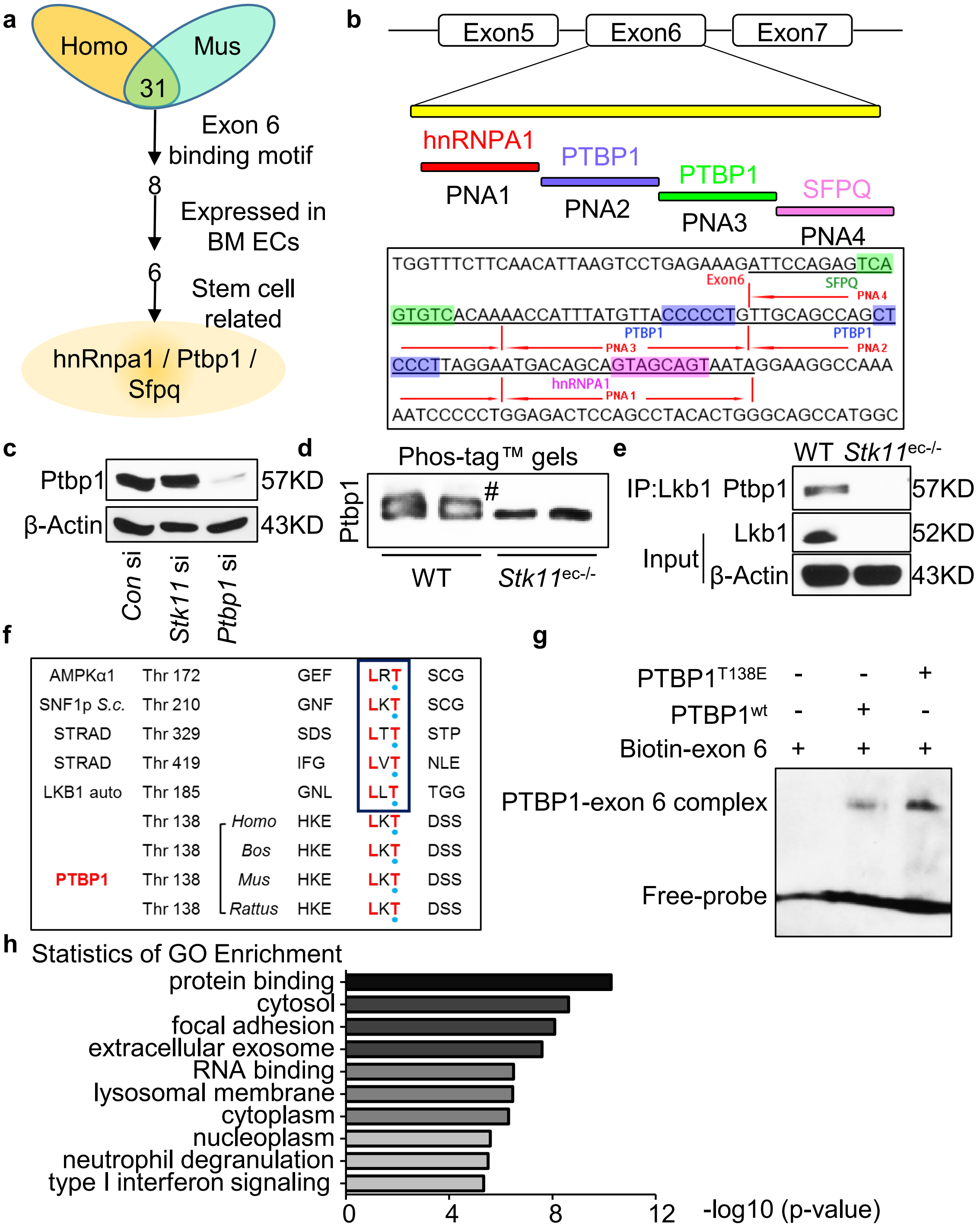

Supplementary Figure 11. Lkb1 phosphorylated Ptbp1. (a) Bioinformatic analysis of potential targeted RNA splicing protein. **(b)** The scheme shows the targeted sequence of PNAs 1-4 in *Scf* exon 6. **(c)** Western blot analysis of *Ptbp1* in *control* or *Stk11*-silenced primary mouse bone marrow ECs. Blots are representative of three independent experiments. **(d)** Phos-tag^{TM} analysis of Ptbp1 in primary mouse bone marrow ECs isolated from WT or *Stk11*$^{ec-/-}$ mice. Blots are representative of three independent experiments. # marked the phosphorylated band. **(e)** Co-immunoprecipitation of Lkb1 with Ptbp1 in primary bone marrow ECs isolated from WT or *Stk11*$^{ec-/-}$ mice. Blots are representative of three independent experiments. **(f)** Bioinformatic analysis of the potential site of Ptbp1 phosphorylation by Lkb1. **(g)** EMSA analysis with cell lysate from Ptbp1^{wt} or Ptbp1^{T138E} overexpressed ECs and biotin-labeled-*Scf (*exon 6) probes. Blots are representative of three independent experiments. **(h)** Gene ontology (GO) analysis of the identified differentially expressed genes among ECs overexpressed with Ptbp1^{wt}, Ptbp1^{T138A} or Ptbp1^{T138E} mutant. The ten most significantly enriched pathways ($P < 0.05$ by Fisher's exact test) were shown.

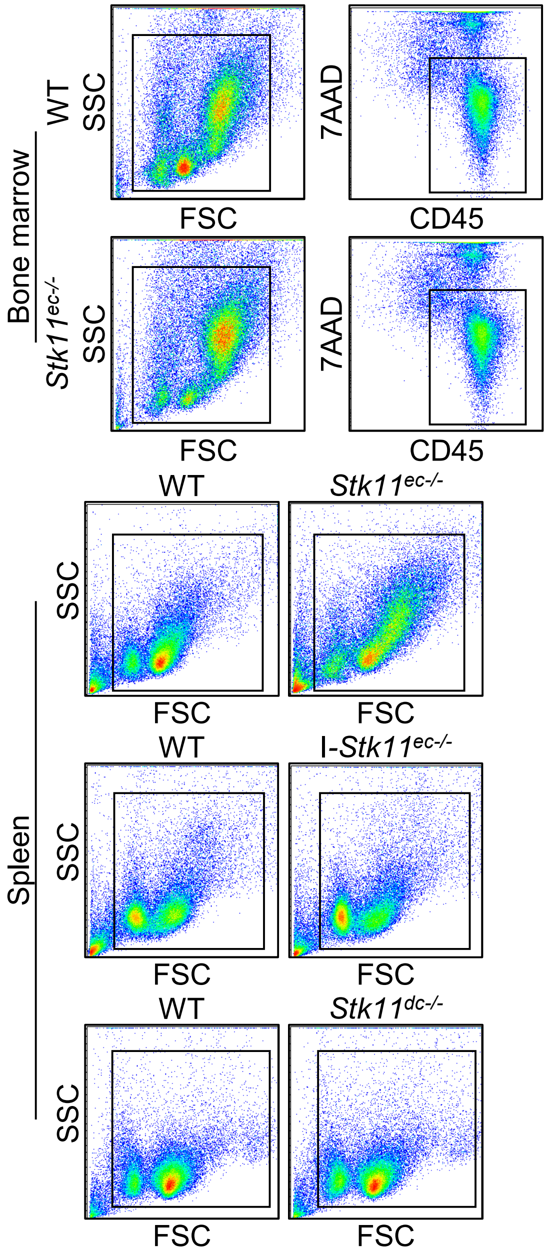

Supplementary Figure 12. Additional gates. Representative images of flow cytometry analysis on spleen and bone marrow from WT or *Stk11*$^{ec-/-}$/I-*Stk11*$^{ec-/-}$/*Stk11*$^{dc-/-}$mice.

Supplementary Table 1. Tumor development in *Stk11*$^{ec-/-}$ mice.

	Genotype	Age (Months)	Organ	Primary Diagnosis	BW (g)	TW (g)	Tumor cell type
1	*Stk11*$^{ec-/-}$	14	Bone	Leukemia	32.51		Leukemia cell
2	*Stk11*$^{ec-/-}$	7	Back	Squamous Carcinoma	37.34	2.24	Epithelial cell
3	*Stk11*$^{ec-/-}$	20	Abdominal	Squamous Carcinoma	38.21	6.39	
4	*Stk11*$^{ec-/-}$	13	Lung	Adenocarcinoma	29.81	1.43	Glandular cells
5	*Stk11*$^{ec-/-}$	14	Uterus	Squamous Carcinoma	30.49	0.61	
6	*Stk11*$^{ec-/-}$	9	Uterus	Endometrial Carcinoma	25.55	0.77	Endometrium
7	*Stk11*$^{ec-/-}$	12	Lung & Uterus	Adenocarcinoma & Endometrial Carcinoma	22.93	0.63 & 1.31	Glandular cells & Endometrium
8	*Stk11*$^{ec-/-}$	18	Abdominal	Lymphoma	28.23	3.72	Lymphocyte
9	*Stk11*$^{ec-/-}$	16	Lung	Carcinoma	27.54	1.26	Epithelial cell
10	*Stk11*$^{ec-/-}$	6	Kidney	Adenocarcinoma	31.81	1.02	
11	*Stk11*$^{ec-/-}$	15	Lung	Adenocarcinoma	21.39	1.26	Glandular cells
12	*Stk11*$^{ec-/-}$	23	Liver	Papillary carcinoma	26.32	0.74	Hepatocyte
13	*Stk11*$^{ec-/-}$	15	Uterus	Endometrial Carcinoma	23.61	0.97	Gland cell
14	*Stk11*$^{ec-/-}$	17	Kidney		28.07	1.31	
15	*Stk11*$^{ec-/-}$	26	Stomach	Adenocarcinoma	31.42		Gland cell
16	*Stk11*$^{ec-/-}$	14	Lung	Adenocarcinoma	27.18	1.20	Glandular cells
17	*Stk11*$^{ec-/-}$	17	Lung	Adenocarcinoma	26.67	0.86	Glandular cells
18	*Stk11*$^{ec-/-}$	19	Abdominal	Lymphoma	26.66	1.06	Lymphocyte
19	*Stk11*$^{ec-/-}$	17	Abdominal	Lymphoma	36.85	1.58	Lymphocyte
20	*Stk11*$^{ec-/-}$	23	Lung	Adenocarcinoma	33.36	0.43	Glandular cells
21	*Stk11*$^{ec-/-}$	15	Skin	Squamous Carcinoma	30.24		Epithelial cell
22	*Stk11*$^{ec-/-}$	11	Intestine	Lymphoma	30.88	1.65	Lymphocyte
23	*Stk11*$^{ec-/-}$	9	Uterus	Leiomyoma	25.85	1.98	Fibroblasts
24	*Stk11*$^{ec-/-}$	12	Abdominal	Lymphoma	34.83	2.87	Lymphocyte
25	*Stk11*$^{ec-/-}$	6	Abdominal	Stromal Tumors	35.06	1.42	Interstitial cell
26	*Stk11*$^{ec-/-}$	11	Abdominal	Lymphoma	29.37	0.99	Lymphocyte

Continued Supplementary Table 1.

	Genotype	Age (Months)	Organ	Primary Diagnosis	BW (g)	TW (g)	Tumor cell type
27	*Stk11*$^{ec-/-}$	27	lung	Carcinoma	27.33	1.34	Epithelial cell
28	*Stk11*$^{ec-/-}$	27	Uterus	Endometrial Carcinoma	24.19	0.69	Endometrium
29	*Stk11*$^{ec-/-}$	22	Liver	Carcinoma	26.41	2.06	Hepatocyte
30	*Stk11*$^{ec-/-}$	18	Bone	Leukemia	23.79		Leukemia cell
31	*Stk11*$^{ec-/-}$	16	Abdominal	Lymphoma	21.09	1.48	Lymphocyte
32	*Stk11*$^{ec-/-}$	17	Kidney	Adenocarcinoma	24.32	2.12	Gland cell
33	*Stk11*$^{ec-/-}$	19	Lung	Adenoma	28.61	1.38	Gland cell

Detailed information for *Stk11*$^{ec-/-}$ mice (*Stk11*$^{fl/fl}$*Cdh5*^{Cre}) with spontaneous tumor development. Age (Months); BW (Body weight, g); TW (Tumor weight, g).

Supplementary Table 2. Tumor development in *Stk11*$^{ec\ wt/-}$ mice.

	Genotype	Age (Months)	Organ	Primary Diagnosis	BW (g)	TW (g)	Tumor cell type
1	*Stk11*$^{ec\ wt/-}$	15	Skin	Squamous Carcinoma	28.21	0.32	Epithelial cell
2	*Stk11*$^{ec\ wt/-}$	20	Abdominal	Lymphoma	27.14	1.32	Lymphocyte
3	*Stk11*$^{ec\ wt/-}$	26	Lung	Adenocarcinoma	32.01	3.42	Gland cell
4	*Stk11*$^{ec\ wt/-}$	22	Abdominal	Lymphoma	27.26	2.75	Lymphocyte
5	*Stk11*$^{ec\ wt/-}$	28	Intestine	Adenocarcinoma	27.86	1.32	Gland cell
6	*Stk11*$^{ec\ wt/-}$	23	Lung	Adenocarcinoma	30.31	2.03	Gland cell

Detailed information for *Stk11*$^{ec\ wt/-}$ mice (*Stk11*$^{fl/wt}$ *Cdh5*^{Cre}) with spontaneous tumor development. Age (Months); BW (Body weight, g); TW (Tumor weight, g).

Supplementary Table 3. Tumor incidence in *Stk11*$^{ec\ wt/-}$ and *Stk11*$^{ec-/-}$ mice.

	***Stk11*$^{ec\ wt/-}$**			***Stk11*$^{ec-/-}$**		
	14 Month	24 Month	30 Month	14 Month	24 Month	30 Month
**NO. examined**	38	19	8	68	23	7
**NO. with tumors**	0	4	2	13	17	3
**Tumor incidence**	0%	21.05%	25.00%	19.12%	73.91%	42.86%
**Lung (Total)**	0	1	1	2	5	1
Adenocarcinoma	0	1	1	2	3	0
Carcinoma	0	0	0	0	1	1
Adenoma	0	0	0	0	1	0
**Abdominal (Total)**	0	2	1	5	9	1
Squamous Carcinoma	0	0	0	0	1	0
Lymphoma	0	2	0	3	4	0
Stroma tumor	0	0	0	1	0	0
Intestine, Adenocarcinoma	0	0	1	0	0	0
Stomach, Adenocarcinoma	0	0	0	0	0	1
Kidney, Adenocarcinoma	0	0	0	1	1	0
Kidney, Carcinoma	0	0	0	0	1	0
Liver, Papillary carcinoma	0	0	0	0	2	0
**Hematopoietic system (Total)**	0	0	0	1	1	0
Lymphoma	0	0	0	0	0	0
Leukemia	0	0	0	1	1	0
**Skin/subcutis (Total)**	0	1	0	1	1	0
Squamous Carcinoma	0	1	0	1	1	0
**Reproductive system (Total)**	0	0	0	3	1	1
Uterus, Squamous Carcinoma	0	0	0	1	0	0
Uterus, Endometrial Carcinoma	0	0	0	1	1	1
Uterus, Leiomyoma	0	0	0	1	0	0
**Multiple systerm (Total)**	0	0	0	1	0	0
Lung, Adenocarcinoma & Uterus, Endometrial Carcinoma	0	0	0	1	0	0

Supplementary Table 4. Genotyping primers.

Mice line	Primers sequence	Type	Size
Stk11^{flox}	5'-TCTAACAATGCGCTCATCGTCATCCTCGGC-3'	Mutant	300 bp
	5'-GAGATGGGTACCAGGAGTTGGGGCT-3'		
	5'-GGGCTTCCACCTGGTGCCAGCCTGT-3'	Wild type	220 bp
	5'-GAGATGGGTACCAGGAGTTGGGGCT-3'		
Cdh5^{Cre}	5'-AGGCAGCTCACAAAGGAACAAT-3'	Transgene	300 bp
	5'-TCGTTGCATCGACCGGTAA-3'		
	5'-CTAGGCCACAGAATTGAAAGATCT-3'	Wild type	324 bp
	5'-GTAGGTGGAAATTCTAGCATCATCC-3'		
ROSA^{EYFP}	5'-AAGACCGCGAAGAGTTTGTC-3'	Mutant	320 bp
	5'-AAAGTCGCTCTGAGTTGTTAT-3'		
	5'-GGAGCGGGAGAAATGGATATG-3'	Wild type	600 bp
	5'-AAAGTCGCTCTGAGTTGTTAT-3'		
Cre	5'-ACTAAACTGGTCGAGCGAGGA-3'		220 bp
	5'-TGTCCAGACCAGGCCAGGTA-3'		

Supplementary Table 5. PCR primers.

Primer	Species		Sequence	Size
(s) S*cf* & (m) S*cf*	*Mus*	F	5'-TCCGAAGAGGCCAGAAACTA-3'	368 & 284 bp
		R	5'-CAACTGCCCTTGTAAGACTTGA-3'	
(s) S*cf*	*Homo*	F	5'-TTCTGAAGGCTTGAGTAATTA-3'	328 bp
		R	5'-ACTGCTACTGCTGTCATTCC-3'	
	Mus	F	5'-CTGAAGGCTTGAGTAATTAC-3'	326 bp
		R	5'-ACTGCTACTGCTGTCATTCC-3'	
	Bos	F	5'-TTCTGAAGGCTTGAGTAATTA-3'	331 bp
		R	5'-ACTGCTACTGCTGTCATTCC-3'	
(m) S*cf*	*Homo*	F	5'-TTCTGAAGGCTTGAGTAATTA-3'	257 bp
		R	5'-TGGCCTTCCCTTTCTCAG-3'	
	Mus	F	5'-CTGAAGGCTTGAGTAATTAC-3'	255 bp
		R	5'-CGGCTTTCCCTTTCTCGG-3'	
	Bos	F	5'-TTCTGAAGGCTTGAGTAATTA-3'	260 bp
		R	5'-AGGCCTTCCCTTTTTCAG-3'	
Gapdh	*Homo*	F	5'-GAGTCAACGGATTTGGTCGT-3'	95 bp
		R	5'-AATGAAGGGGTCATTGATGG-3'	
	Mus	F	5'-AAGGTCATCCCAGAGCTGAA-3'	222 bp
		R	5'-CTGCTTCACCACCTTCTTGA-3'	

Supplementary Table 6. Mutation primers.

Primer	Sequence	
(h) Ptbp1-T138A	F	5'-CCGACAGCTCTCCCAACCAGGC-3'
(h) Ptbp1-T138E	F	5'-GAAGACAGCTCTCCCAACCAGGC-3'
(h) Ptbp1-T138-Common	R	5'-CTTCAGCTCCTTGTGGTTGG-3'

Supplementary Table 7. Antibody

Experiment	Antibody	Catalog number	Dilution
**Two-photon deep imaging of bone marrow**	GFP	Aves Labs, GFP-1020	1:200
	c-Kit	R&D, #BAF1356	1:100
	laminin	Abcam, #ab7463	1:200
	CD16/32	BioLegend, #101302	1:100
**Western blotting**	Ptbp1	CST, #57246	1:1000
	Lkb1	Santa Cruz, #32245	1:1000
	β-Actin	Santa Cruz, #47778	1:1000
	Gapdh	Santa Cruz, #32233	1:1000
	His	CST, #2365	1:1000
**IF**	vWF	Abcam, #ab11713	1:100
	LKB1	LSBio, #LS-B6669	1:100
**IHC**	LKB1	Lifespan, #LS-B11921	1:100
	CD61	Novus, #NBP1-83453	1:200

Continued Supplementary Table 7.

Experiment	Antibody	Catalog number	Dilution
**FCM**	CD11c	BioLegend, #117307	1:100
	MHC class II	BioLegend, #107635	1:100
	B220	BioLegend, #103255	1:100
	B220	BD, #563893	1:100
	B220	BioLegend, #103227	1:50
	CD11b	BioLegend, #101245	1:50
	CD11b	BioLegend, #101263	1:50
	CD45	BioLegend, # 103155	1:50
	SIRPα	BioLegend, #144008	1:100
	Flt3	BioLegend, #135306	1:200
	c-Kit	BioLegend, #135125	1:50
	CD115	BioLegend, #135524	1:200
	CX3CR1	BioLegend, #149025	1:2000
	CD3	BioLegend, #100334	1:50
	CD19	BioLegend, #115523	1:200
	CD49b	BioLegend, #108918	1:50
	Ly6G	BioLegend, #127612	1:50
	CD103	BioLegend, #121406	1:100
	CD64	BioLegend, #139309	1:40
	NK1.1	BioLegend, #108722	1:50
	Ter119	BioLegend, #116232	1:200
	F4/80	BioLegend, #123133	1:50
