# Supplementary material for: Endothelial cell-specific expression of serine/threonine kinase 11 modulates dendritic cell differentiation
Source: Nat Commun. 2022 Feb 3;13:648. doi: 10.1038/s41467-022-28316-6 (PMC8814147; doi:10.1038/s41467-022-28316-6)
Supplement: Supplementary file 2 — Description of Additional Supplementary Files [file 41467_2022_28316_MOESM2_ESM.pdf]

Description of Additional Supplementary Files

File Name: Supplementary Movie 1
Description: Video of deep imaging of *Cx3cr1*-GFP$^+$ cells (Green), hematopoietic progenitors (c-Kit$^+$, red), and blood vessels (Laminin, Gray) in digitally reconstructed bone marrow (300 µm thick).
