# Supplementary material for: Endothelial cell-specific expression of serine/threonine kinase 11 modulates dendritic cell differentiation
Source: Nat Commun. 2022 Feb 3;13:648. doi: 10.1038/s41467-022-28316-6 (PMC8814147; doi:10.1038/s41467-022-28316-6)
Supplement: Supplementary file 4 — Reporting Summary [file 41467_2022_28316_MOESM4_ESM.pdf]

nature research

Corresponding author(s): Ming-hui Zou
Last updated by author(s): Sep 22, 2021

Reporting Summary

Nature Research wishes to improve the reproducibility of the work that we publish. This form provides structure for consistency and transparency in reporting. For further information on Nature Research policies, see our Editorial Policies and the Editorial Policy Checklist.

Statistics

For all statistical analyses, confirm that the following items are present in the figure legend, table legend, main text, or Methods section.

n/a	Confirmed	
☐	☒	The exact sample size (n) for each experimental group/condition, given as a discrete number and unit of measurement
☐	☒	A statement on whether measurements were taken from distinct samples or whether the same sample was measured repeatedly
☐	☒	The statistical test(s) used AND whether they are one- or two-sided<br>*Only common tests should be described solely by name; describe more complex techniques in the Methods section.*
☐	☒	A description of all covariates tested
☐	☒	A description of any assumptions or corrections, such as tests of normality and adjustment for multiple comparisons
☐	☒	A full description of the statistical parameters including central tendency (e.g. means) or other basic estimates (e.g. regression coefficient) AND variation (e.g. standard deviation) or associated estimates of uncertainty (e.g. confidence intervals)
☐	☒	For null hypothesis testing, the test statistic (e.g. F, t, r) with confidence intervals, effect sizes, degrees of freedom and P value noted<br>*Give P values as exact values whenever suitable.*
☒	☐	For Bayesian analysis, information on the choice of priors and Markov chain Monte Carlo settings
☒	☐	For hierarchical and complex designs, identification of the appropriate level for tests and full reporting of outcomes
☐	☒	Estimates of effect sizes (e.g. Cohen's d, Pearson's r), indicating how they were calculated

Our web collection on statistics for biologists contains articles on many of the points above.

Software and code

Policy information about availability of computer code

Data collection	FCM: BD LSRFortessa™ Flow Cytometer; Western blot: Kodak X-Omat 2000A; DNA sequence: Thermo Fisher Scientific DNA Sequencer 3730; PCR: BIO-RAD C1000 Touch Thermal Cycler; Rt-PCR: CFX96 Touch Deep Well Real-Time PCR System; Cell counter: Vi-Cell cell counter Beckman Coulter; Microscope: OLYMPUS BX51 fluorescence microscope; 3D Imaging: Zeiss 710 & Leica SP8 MP.
Data analysis	Western blot: Image J; FCM: FlowJo; Statistics: SPSS; Graph: GraphPad Prism 5.0.

For manuscripts utilizing custom algorithms or software that are central to the research but not yet described in published literature, software must be made available to editors and reviewers. We strongly encourage code deposition in a community repository (e.g. GitHub). See the Nature Research guidelines for submitting code & software for further information.

Data

Policy information about availability of data

All manuscripts must include a data availability statement. This statement should provide the following information, where applicable:
- Accession codes, unique identifiers, or web links for publicly available datasets
- A list of figures that have associated raw data
- A description of any restrictions on data availability

Source data are provided with this paper.

Field-specific reporting

Please select the one below that is the best fit for your research. If you are not sure, read the appropriate sections before making your selection.

☒ Life sciences ☐ Behavioural & social sciences ☐ Ecological, evolutionary & environmental sciences

For a reference copy of the document with all sections, see nature.com/documents/nr-reporting-summary-flat.pdf

Life sciences study design

All studies must disclose on these points even when the disclosure is negative.

Sample size	Software G Power (Faul, Erdfelder, Lang and Buchner, 2007) was used for sample size calculation.
Data exclusions	No data were excluded.
Replication	Each western blot and PCR were repeated at least three independent times. Animal experiment were repeated based on statistic requirement.
Randomization	Simple Random Sampling. Basically, assign numbers to participants, or treatments, and use a random number table to choose participants and treatment groups.
Blinding	The investigators were blinded to group allocation during data collection and analysis.

Reporting for specific materials, systems and methods

We require information from authors about some types of materials, experimental systems and methods used in many studies. Here, indicate whether each material, system or method listed is relevant to your study. If you are not sure if a list item applies to your research, read the appropriate section before selecting a response.

Materials & experimental systems

n/a	Involved in the study	
☐	☒	Antibodies
☐	☒	Eukaryotic cell lines
☒	☐	Palaeontology and archaeology
☐	☒	Animals and other organisms
☒	☐	Human research participants
☒	☐	Clinical data
☒	☐	Dual use research of concern

Methods

n/a	Involved in the study	
☒	☐	ChIP-seq
☐	☒	Flow cytometry
☒	☐	MRI-based neuroimaging

Antibodies

Antibodies used	CD11c (BioLegend, #117307), MHC class II (BioLegend, #107635), CD11c (BioLegend, #117307), B220 (BioLegend, #103255), CD11b (BioLegend, #101239), Lineage Cocktail (BioLegend, #133306), SIRPα (BioLegend, #144008), Flt3 (BioLegend, #135306),c-Kit (BioLegend, #135125), anti-CD115 (BioLegend, #135524), CX3CR1 (BioLegend, #149025), Flt3 (BioLegend, #135306) antibodies were used for FCM.<br>Von Willebrand factor (Abcam, #ab11713), CD11c (Thermo Fisher, #14-0114-85), Lkb1 (Lifespan, #LS-B11921) and CD61 (Novus, #NBP1-83453) antibodies were used for staining.<br>Lkb1 (#sc-32245, 1:1000), β-Actin (, #sc-47778, 1:1000) and Gapdh (#sc-47724, 1:1000) antibodies were used for western blot.<br>GFP (Aves Labs, GFP-1020), c-Kit (R&D Systems, #BAF1356), and laminin (Abcam, #ab7463) antibodies were used for 3D staining.
Validation	Anti-CD11c (BioLegend, #117307) and anti-MHC class II (BioLegend, #107635) antibodies were used to stain classical DCs. Anti-CD11c (BioLegend, #117307) and anti- B220 (BioLegend, #103255) were used to stain plasmacytoid DCs. Anti-CD11c (BioLegend, #17307) and anti- CD11b (BioLegend, #101239) were used to stain macrophages. Anti-Lineage Cocktail (BioLegend, #133306), anti-CD11c (BioLegend, #117307), anti-MHC class II (BioLegend, #107635), anti-SIRPα (BioLegend, #144008) and anti-Flt3 (BioLegend, #135306) were used to stain pre-classical DCs. Anti-Lineage Cocktail (BioLegend, #133306), anti-c-Kit (BioLegend, #135125), anti-CD115 (BioLegend, #135524), anti-CX3CR1 (BioLegend, #149025), and anti-Flt3 (BioLegend, #135306) were used to stain monocyte and dendritic cell progenitor (MDP). Anti-Lineage Cocktail (BioLegend, #133306), anti-c-Kit (BioLegend, #135125), anti-CD115 (BioLegend, #135524), and anti-Flt3 (BioLegend, #135306) were used to stain common DC progenitors.<br>Von Willebrand factor (Abcam, #ab11713), CD11c (Thermo Fisher, #14-0114-85), Lkb1 (Lifespan, #LS-B11921) and CD61 (Novus, #NBP1-83453) antibodies were used for staining.<br>Lkb1 (#sc-32245, 1:1000), β-Actin (, #sc-47778, 1:1000) and Gapdh (#sc-47724, 1:1000) antibodies were used for western blot.<br>GFP (Aves Labs, GFP-1020), c-Kit (R&D Systems, #BAF1356), and laminin (Abcam, #ab7463) antibodies were used for 3D staining.

Eukaryotic cell lines

Policy information about cell lines

Cell line source(s)	Primary bone marrow cells were collected from indicated mice. AFT024 bought from ATCC® SCRC-1007™.
Authentication	Bone marrow cells were authenticated by FCM, through staining of EC specific markers. AFT024 cells were confirmed by cell culture and morphology.
Mycoplasma contamination	Cells test negative for mycoplasma contamination
Commonly misidentified lines (See ICLAC register)	N/A

Animals and other organisms

Policy information about studies involving animals; ARRIVE guidelines recommended for reporting animal research

Laboratory animals	Stk11fl/fl, Cdh5Cre, Cx3cr1Cre, Cx3cr1GFP, Gt (ROSA)26Sortm1(EYFP)Cos/J mice were obtained from The Jackson Laboratory. VECad-CreERT2 mice were provided by Dr. Xin Zhang (University of Oklahoma Health Science Center). All experiment animals used are mixed age and sex, if not specific delineated in the paper.
Wild animals	N/A
Field-collected samples	All mice were kept in a controlled temperature (21.8 ± 0.7°C) and humidity (49.16 ± 2.37%) environment with a 12-h light/dark cycle and fed a rodent diet with free access to water.
Ethics oversight	All animal protocols were approved by the Georgia State University Committee on the Use and Care of Animals.

Note that full information on the approval of the study protocol must also be provided in the manuscript.

Flow Cytometry

Plots

Confirm that:

☒ The axis labels state the marker and fluorochrome used (e.g. CD4-FITC).

☒ The axis scales are clearly visible. Include numbers along axes only for bottom left plot of group (a 'group' is an analysis of identical markers).

☒ All plots are contour plots with outliers or pseudocolor plots.

☒ A numerical value for number of cells or percentage (with statistics) is provided.

Methodology

Sample preparation	Primary bone marrow cells were collected from indicated mice. AFT024 bought from ATCC® SCRC-1007™. Stk11fl/fl, Cdh5Cre, Cx3cr1Cre, Cx3cr1GFP, Gt (ROSA)26Sortm1(EYFP)Cos/J mice were obtained from The Jackson Laboratory. VECad-CreERT2 mice were provided by Dr. Xin Zhang (University of Oklahoma Health Science Center).
Instrument	FCM: BD LSRFortessa™ Flow Cytometer; Western blot: Kodak X-Omat 2000A; DNA sequence: Thermo Fisher Scientific DNA Sequencer 3730; PCR: BIO-RAD C1000 Touch Thermal Cycler; Rt-PCR: CFX96 Touch Deep Well Real-Time PCR System; Cell counter: Vi-Cell cell counter Beckman Coulter; Microscope: OLYMPUS BX51 fluorescence microscope; 3D Imaging: Zeiss 710 & Leica SP8 MP.
Software	BD LSRFortessa™ Flow Cytometer is used to collect FCM data. FlowJo is used to analyze FCM data.
Cell population abundance	Primary bone marrow endothelial cells were used only for generation. After collecting, the cell purity was confirmed by FCM. The purity is higher than 90%.
Gating strategy	The first step in gating is often distinguishing populations of cells based on their forward and side scatter properties. After remove dead cells, exclude populations (e.g. debris) or include positively select populations for further examination. Isotype, FMO and unstained controls are included to accurately identify the positive dataset. When doing multiple-parameter density plots, gates were placed around the distinct populations. We also use backgating to confirm a staining pattern or gating method.

☒ Tick this box to confirm that a figure exemplifying the gating strategy is provided in the Supplementary Information.
